# Sex and Gender Driven Modifiers of Alzheimer’s: The Role for Estrogenic Control Across Age, Race, Medical, and Lifestyle Risks

**DOI:** 10.3389/fnagi.2019.00315

**Published:** 2019-11-15

**Authors:** Aneela Rahman, Hande Jackson, Hollie Hristov, Richard S. Isaacson, Nabeel Saif, Teena Shetty, Orli Etingin, Claire Henchcliffe, Roberta Diaz Brinton, Lisa Mosconi

**Affiliations:** ^1^Department of Neurology, Weill Cornell Medicine, Cornell University, New York, NY, United States; ^2^Concussion Clinic, Hospital for Special Surgery, New York, NY, United States; ^3^Department of Internal Medicine, Weill Cornell Medicine, Cornell University, New York, NY, United States; ^4^Department of Pharmacology, College of Medicine, The University of Arizona, Tucson, AZ, United States; ^5^Department of Neurology, College of Medicine, The University of Arizona, Tucson, AZ, United States; ^6^Department of Radiology, Weill Cornell Medicine, Cornell University, New York, NY, United States; ^7^Department of Psychiatry, New York University School of Medicine, New York, NY, United States

**Keywords:** Alzheimer’s disease, estrogen hypothesis, sex differences, gender differences, menopause transition, risk factors

## Abstract

Research indicates that after advanced age, the major risk factor for late-onset Alzheimer’s disease (AD) is female sex. Out of every three AD patients, two are females with postmenopausal women contributing to over 60% of all those affected. Sex- and gender-related differences in AD have been widely researched and several emerging lines of evidence point to different vulnerabilities that contribute to dementia risk. Among those being considered, it is becoming widely accepted that gonadal steroids contribute to the gender disparity in AD, as evidenced by the “estrogen hypothesis.” This posits that sex hormones, 17β-estradiol in particular, exert a neuroprotective effect by shielding females’ brains from disease development. This theory is further supported by recent findings that the onset of menopause is associated with the emergence of AD-related brain changes in women in contrast to men of the same age. In this review, we discuss genetic, medical, societal, and lifestyle risk factors known to increase AD risk differently between the genders, with a focus on the role of hormonal changes, particularly declines in 17β-estradiol during the menopause transition (MT) as key underlying mechanisms.

## Introduction

Alzheimer’s disease (AD) is a neurodegenerative disease marked by impairments in memory, attention, language, and daily living activities (Alzheimer’s Association, 2017). While AD currently impacts 5.7 million Americans regardless of ethnic and cultural backgrounds (Alzheimer’s Association, 2017), the prevalence is expected to triple by 2050, with nearly 14 million patients affected. Similar trends have been reported worldwide with a projected 130 million patients in the next 30 years.

Alzheimer’s disease is an extremely debilitating condition currently falling within the top 10 causes of death across the world. This causes a severe fiscal burden on health services since AD is an extremely financially costly neurological disease to manage (Nichols et al., 2019). Addressing the economic and social costs of AD is increasing in urgency as the Baby Boomer generation ages and life expectancy increases. Recent studies estimate that, from 2010 to 2050, annual costs will increase from $307 billion to $1.5 trillion in the United States alone (Zissimopoulos et al., 2015). Medical advances that delay disease onset for 5 years or longer would result in a 41% lower prevalence and 40% lower cost of AD in 2050 (Zissimopoulos et al., 2015).

To date, there has been a lack of therapeutics to prevent, delay, or reverse late-onset AD, resulting in a host of unsuccessful clinical trials. Research efforts over the past decade have prioritized therapeutic strategies that aim to remove beta-amyloid (Aβ) and tau pathology or prevent their accumulation, with limited success (Andrieu et al., 2015). Therefore, there exists an urgent and unmet need to develop novel strategies to prevent dementia, or at the very least delay its onset, or slow down progression. Several reasons underlie these past failures; among the most far-reaching are the stage at which therapeutic interventions are initiated, and the sex differences in the underlying mechanisms leading to AD.

It has become widely accepted that the pathophysiological mechanisms of AD begin decades before the emergence of clinically detectable symptoms and contribute to a 15–20 year’s prodromal or “preclinical” disease stage starting in midlife (Sperling et al., 2014). Failure to develop successful disease-modifying therapies may be because the majority of interventions have been tested in cohorts with clinically manifest disease and thus substantial synaptic and neuronal damage. Initiating therapies during the preclinical phase of AD will likely yield greater chances of success, a recognition that has effectively paved the way for primary and secondary AD prevention trials (Andrieu et al., 2015).

There is also emerging evidence that several medical, environmental, and lifestyle risk factors that lead to AD development are modifiable (Livingston et al., 2017). At least one out of three AD dementia cases can be linked to medical factors such as cardiovascular conditions, obesity, diabetes, and lifestyle factors such as physical activity, diet, social engagement, and educational attainment (Norton et al., 2014). Until disease-modifying treatment becomes available, risk reduction interventions could still drastically reduce the future burden of AD at the population level (Isaacson et al., 2018).

In this context, it is being widely accepted that many of the above AD risk factors show gender effects, with female sex being more severely impacted (Ferretti et al., 2018; Nebel et al., 2018; Scheyer et al., 2018). It has long been known that, after advanced age, female sex is the major risk factor for AD (Farrer et al., 1997). Currently, two-thirds of AD patients are females. Postmenopausal women comprise over 60% of those patients (Brookmeyer et al., 1998). Increasing effort has thus been devoted to identifying sex-specific differences in disease etiology, manifestation, and progression as a crucial step toward gender-based disease prevention. Among putative biological mechanisms, it is becoming widely accepted that gonadal steroids contribute to the gender disparity in AD, as evidenced by the “estrogen hypothesis” presented herein. This posits that female sex hormones, 17β-estradiol in particular, exert a neuroprotective effect by buffering females’ brains against disease development. Hormonal changes in the years leading up to and after menopause are linked to the emergence of AD-related brain changes in females in contrast to males of the same age. In this review, we provide a comprehensive review of genetic, medical, societal, and lifestyle risk factors known to increase AD risk differently between the genders, with a focus on the role of gonadal hormones as key underlying mechanisms.

## The Estrogen Hypothesis

It is has long been proposed that gonadal steroids contribute to gender differences in AD. Several reproductive hormones and their interactions may be implicated, including estrogen, progesterone, luteinizing hormone, and follicle stimulating hormones. All these so-called female hormones naturally fluctuate over endogenous hormonal cycles. Nonetheless, this review will focus primarily on estrogen since considerable evidence from molecular, animal, and clinical studies indicates that, of all gonadal hormones, estrogen may be particularly involved in the pathophysiology of AD-dementia in women. The “estrogen hypothesis” postulates that estrogen plays a protective role against AD-dementia, while that estrogen dysfunction seems to exacerbate, or perhaps precipitate the AD process in women.

Even though it is present in both sexes, estrogen is often considered the primary female sex hormone. Reference to estrogen broadly refers to numerous compounds such as estrone (E_1_), estradiol (E_2_), and estriol (E_3_). The primary circulating estrogen during a woman’s reproductive years is 17β-estradiol, which is also the strongest form. For the purposes of this review, estrogen refers to 17β-estradiol, the endogenous form. 17β-estradiol plays a role in the formation of secondary sex characteristics in females and reproduction in males, and has peripheral effects in the liver and bone in both sexes (Cui et al., 2013). While it is primarily central to the ovaries for menstrual cycle coordination in women, it is also made by non-endocrine tissues, such as fat, breasts, and the brain (McEwen et al., 1997).

**FIGURE 1:**
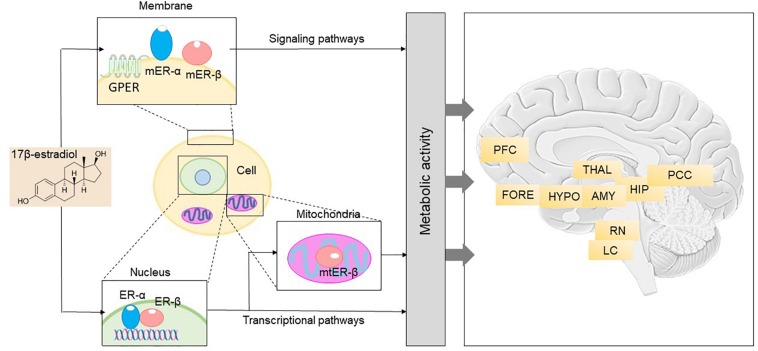
Brain 17β-estradiol receptor network and anatomical distribution (Adapted with permission from Brinton et al., 2015). Right: 17β-estradiol receptor network in the brain includes different pathways. The binding of estrogen and consequent activation of the membrane and trans-membrane receptors, mER-α, mER-β, and GPER, contributes to initiation of signaling networks that mediate early and intermediate gene expression response. Binding of estrogen to ER-α and ER-β, the nuclear estrogen receptors, leads to initiation of transcriptional pathways that also regulate late response gene expression. Activation and translocation of ER-β to the mitochondria has been implicated in expression of mitochondrial genes. Furthermore, estrogen can modulate transcriptional gene expression via epigenetic regulation. This integrated network of receptors enables coordination of a broad spectrum of cellular elements, which ultimately results in generation of energy to fuel neurological function. ER, estrogen receptor; GPER, G-protein coupled estrogen receptor 1; mER, membrane estrogen receptor; mtER, mitochondrial estrogen receptor. Left: Anatomical basis for the neurological symptoms that can emerge during the menopause transition. Nuclear, membrane-associated, and mitochondrial estrogen receptors are distributed within each of the neural circuits depicted and can be present in both neurons and glial cells. Dysregulation of estrogen signaling and transcriptional pathways, either through changes in estrogen concentration or through modifications of estrogen receptor activity, impacts neurological function in those areas. AMY, amygdala; FORE, basal forebrain; HIP, hippocampus; HYPO, hypothalamus; LC, locus coeruleus; PCC, posterior cingulate cortex; PFC, prefrontal cortex; RN, raphe nucleus; THAL, thalamus.

Estrogen affects several areas of the brain, thereby influencing cognitive function, affect, and behavior (Fink et al., 1996; Dumitriu et al., 2010; Brinton et al., 2015). Several lines of research have demonstrated that estrogen is a vital signaling molecule within the brain (Brinton, 2008; Rettberg et al., 2014). It can not only go through the blood–brain barrier but the brain also produces estrogen endogenously from cholesterol (Balthazart and Ball, 2006; Rettberg et al., 2014). Estrogen utilizes a network of receptors and signaling pathways to initiate and regulate molecular and genomic responses required for survival at the level of the cells, genes, organs, and ultimately, the whole body (Figure 1; Rettberg et al., 2014). Estrogen receptors (ERs) are expressed by both sexes and are found on both neurons and glial cells throughout the brain (Rettberg et al., 2014). These receptors are conserved evolutionarily, with homologs present in all vertebrates. There are three types of ERs that have been discovered, to date: estrogen receptor 1 (ESR1 or ERα), estrogen receptor 2 (ESR2 or ERβ), and G-protein coupled estrogen receptor 1 (GPER) (Brinton et al., 2015). Binding of estrogen to these receptors activates several signaling pathways and cellular processes via both genomic and non-genomic processes (Brinton et al., 2015).

Of importance to the brain aging process, estrogen has known neuroprotective properties through its effects on spinogenesis, protecting the brain from age-related and toxic insults. Research using female rats in the early 1990s demonstrated that the density of dendritic spines on the CA1 region neurons of the hippocampus shifts over the ovarian cycle period (Gould et al., 1990) and that surgical oophorectomy, the removal of one or both ovaries, contributes to a 30% loss in spine density that can be recovered neurons by estrogen replacement (Woolley et al., 1990). This estrogen-led spinogenesis is followed by an equal increase in synapses (Woolley and McEwen, 1992) pointing to potential integration of the new spines into the hippocampal network.

Estrogen is also fundamental in metabolic regulation of the brain and body (Brinton et al., 2015). For instance, it regulates glucose transport, aerobic glycolysis, and mitochondrial function to generate ATP in the brain (Rettberg et al., 2014). In animal models, oophorectomy causes a significant reduction in multiple brain glucose transporters, including GLUT-1, GLUT-3, and GLUT-4 (Brinton, 2009). Loss of ovarian hormones with reproductive aging leads to a significant reduction in brain glucose activity, which could be attributed to decreased neuronal glucose transporter expression, compromised hexokinase activity, inactivation of the pyruvate dehydrogenase complex (PDC), and eventually a functionally significant decrease in mitochondrial bioenergetic function (Ding et al., 2013; Rettberg et al., 2014). In addition to facilitating glucose transport, estrogen also promotes neuronal aerobic glycolysis and potentiates mitochondrial bioenergetics through its positive effects on pyruvate dehydrogenase (PDH), aconitase, and ATP synthase (Nilsen et al., 2007; Rettberg et al., 2014).

Estrogen has also been shown to protect DNA against damage induced by hydrogen peroxide (H_2_O_2_) and arachidonic acid by increasing expression of a multitude of antioxidant enzymes, such as glutaredoxin, peroxiredoxin 5, and MnSOD (Nilsen et al., 2007; Rettberg et al., 2014). This estrogen-induced increase in antioxidants subsequently leads to a decrease in free radicals and oxidative damage to mitochondrial DNA and is potentially thought to contribute to the longer life span of women compared to men (Vina et al., 2006).

Overall, these studies highlight the role of estrogen in brain aging and neurodegenerative diseases such as AD. More research is warranted to understand the effect of aging on brain estrogen activity, especially in the context of ERα and ERβ expression and signaling. So far, data suggest that in different parts of the brain, decreased ERα responsiveness may mediate cognitive decline and dementia risk (Yaffe et al., 2009). Although ERβ is at least partially receptive to E_2_ during aging, it may be unable to compensate for the lack of ERα (Foster, 2012). With aging, there is also an increase in particular ERα splice variants in some parts of the brain, especially the hippocampus, that cause most of the available ERα to be non-functional (Ishunina et al., 2007). Interestingly, research has shown that elderly women are more likely to have greater expression of ERα splice variants than elderly men (Foster, 2012; Rettberg et al., 2014). In addition to splice variants, there are numerous ERα polymorphisms that increase AD risk specifically in women, particularly when linked to the APOE ε4 allele (Ryan et al., 2014; Brinton, 2017) which is a major AD genetic risk factor (discussed below).

Further evidence for the “estrogen hypothesis” comes from studies that have implicated the menopause transition (MT) with the emergence of AD-related brain changes in women at risk for developing AD (Brinton et al., 2015). The MT is associated with neurological symptoms such as disturbances of estrogen-regulated thermoregulation, sleep, onset of depression, and cognitive changes and ultimately results in reproductive senescence (Brinton et al., 2015; Figure 1). In the brain, ERs are widely found in the hypothalamic preoptic nucleus which serves as the primary thermoregulatory center; the suprachiasmatic nucleus of the hypothalamus which plays a central role in sleep and circadian rhythms regulation; the 5-hydroxy-tryptaminergic neurons of the raphe nucleus involved in affect and mood; and neurons in the locus coeruleus responsible for arousal and anxiety (McEwen et al., 1997; Brinton, 2017). In brain regions that are important for thinking, learning, and memory, ERs are present in the prefrontal cortex, medial temporal regions such as hippocampus and amygdala, and in the posterior cingulate and retrosplenial cortex (Shughrue et al., 1997; McEwen et al., 2012). It has been proposed that during menopause, decline in circulating estrogen is coincident with decline in brain bioenergetics and shift toward a metabolically compromised phenotype in these brain regions (Rettberg et al., 2014). Inadequate or absent compensatory bioenergetic adaptations to lack of estrogenic activation would then trigger not only the signature symptoms of menopause (hot flashes, night sweats), insomnia, and depressive mood symptoms, but also cognitive changes, thereby increasing risk of late-onset AD in postmenopausal women (Zhao et al., 2016; Bacon et al., 2019).

As later described in more detail, epidemiological data found an increased risk of dementia in women who underwent either unilateral or bilateral oophorectomy (surgical removal of the ovaries) before the onset of natural menopause (Rocca et al., 2007). These findings have been confirmed and extended to include hysterectomy with and without oophorectomy (Phung et al., 2010). Additionally, brain imaging studies of women undergoing natural menopause provided evidence that the MT is related to a greater risk for AD-brain changes in middle-aged peri- and postmenopausal women compared to men of similar age (Mosconi et al., 2017b, 2018). Further, the MT leads to an increased risk of depression, cardiovascular disease (CVD), type 2 diabetes mellitus (T2DM), and metabolic syndrome (MetS) in women (Pucci et al., 2017), as well as a compromised response to head injuries – all of which serve as AD risk factors (Biessels et al., 2006; Livingston et al., 2017). Women exhibit increased vulnerability to a variety of environmental and lifestyle AD risk factors like physical inactivity, an imbalanced diet, disrupted sleep, and chronic stress. These findings highlight the higher susceptibility of women to AD and propose a potential window of opportunity for the implementation of AD risk reduction strategies.

In the end, several lines of research provide support to the hypothesis that repeated hormone influxes in women confer protection against brain aging, while the onset of menopause may exacerbate an existing AD predisposition (Paganini-Hill and Henderson, 1994; Rocca et al., 2007, 2014; Fisher et al., 2018). An increasing number of studies have investigated estrogen therapy to potentially treat AD, as well as for CVD risk reduction in women (Mulnard et al., 2000; Resnick and Henderson, 2002; Maki, 2013). As reviewed below, earlier studies generally showed lack of benefits and even a potential harmful effect, whereas recent re-examinations indicate that the efficacy of estrogen in sustaining neurological health and function depends on several key factors, such as the time of initiation of estrogen therapy, the functioning of the brain at the time of therapy initiation, and the forms of hormones used (Brinton, 2004). Additionally, both pharmacological and non-pharmacological therapies aimed at supporting hormonal levels in aging women may help attenuate the impact of modifiable AD risk factors on the brain.

The aim of this review is to offer an updated review of the literature with respect to female-specific risk factors for AD (summarized in Table 1), and to put forth the estrogen hypothesis as a unifying mechanism of estrogen action on pre-existing and environmental risk. As previously discussed by Nebel et al. (2018), sex points to differences in biology such as chromosomal or hormonal factors, whereas gender refers to differences in the impact of psychosocial, cultural, and environmental influences on biological factors between men and women. Both sex- and gender-related risk factors are included below.

**TABLE 1:** Sex- and gender-related AD risk factors.

		**Risk factor**	**Effect on AD risk**
Sex differences	Genetic risks	APOE epsilon 4 allele	F > M
		Race (black, hispanic)	F > M
	Medical risks	Cardiovascular disease: Microvascular pathology (e.g., coronary microvascular obstruction and endothelial inflammation) Myocardial infarction Stroke (aneurysms)	F > M F > M after menopause F > M after menopause
		Type 2 diabetes; insulin resistance; prediabetes	F > M after menopause
		Depression	F > M after menopause
		Traumatic brain injury; concussions	F > M
		Chronic inflammation	F > M
		Systemic infection	F > M
Female sex-specific	Hormonal risks	Female sex	F only
		Thyroid disease (hyperthyroidism; hypothyroidism)	F > M
		Pregnancy (preeclampsia, gestational diabetes, post-partum depression)	F only
		Menopause (natural menopause; surgically induced menopause)	F only
Gender differences	Lifestyle risks	Educational attainment	May affect F > M
		Occupation	May affect F > M
		Intellectual activity	May affect F > M
		Physical activity	F > M
		Diet	May affect F > M
		Sleep	F > M after menopause
		Stress	F > M after menopause
		Caregiver burden	F > M
		Marital status	M > F

*F, female, M, male.*

### Female Sex

As mentioned above, female sex is a major risk factor for late-onset AD (Farrer et al., 1997). Approximately two-thirds of the more than 5 million Americans affected with AD are women and two-thirds of the more than 15 million Americans caring for someone with AD are women. As such, women find themselves at the epicenter of the impending AD epidemic. As grave a concern as breast cancer is to women’s health, women in their 1960s are almost two times more likely to develop AD over the rest of their lives as they are to develop breast cancer (Alzheimer’s Association, 2017). A woman in her 1960s has an estimated lifetime risk of one in six for developing Alzheimer’s whereas the risk is 1 in 11 for a man of the same age (Alzheimer’s Association, 2017).

The prevailing view holds women’s greater longevity relative to men, which makes women more likely to reach the ages of greater risk, as the main reason for the increased AD prevalence (Seshadri et al., 1997; Hebert et al., 2001). Studies in support of the increased longevity view have focused on estimates of incidence as well as prevalence. While it is well established that the prevalence of AD (i.e., the number of affected patients) is higher in females than in males, it is still not known whether incidence, i.e., the number of people who develop AD during a particular time period, also differs. The few studies investigating this issue found that in Europe and other areas, women also develop AD at a higher rate than men, especially at older ages (Fratiglioni et al., 1997; Ott et al., 1998; Ruitenberg et al., 2001; Prince et al., 2016). However, in the United States, the incidence seems to be similar across both genders (Edland et al., 2002; Miech et al., 2002; Chêne et al., 2015). It is important to recognize that if men and women are developing AD at the same rate, but prevalence is ultimately higher in women, then the higher disease prevalence in women might indeed be attributed to their longer survival rates.

Research on prevalence and incidence rates of mild cognitive impairment (MCI), an intermediary stage between cognitive changes associated with normal aging and dementia, has provided mixed results (Mielke et al., 2014). Some studies report that men have a higher prevalence of MCI (Koivisto et al., 1995; Ganguli et al., 2004) whereas others indicate greater prevalence in women (Larrieu et al., 2002; Di Carlo et al., 2007). In terms of incidence, women tend to show an increased MCI incidence at older ages (Mielke et al., 2014), while men consistently exhibit a higher incidence of the non-amnestic MCI, which is believed to be prodromal for other dementias, such as vascular dementia (Caracciolo et al., 2008; Roberts et al., 2012).

While longevity is an important issue to consider, emerging evidence suggests that there are unique biological reasons for the increased AD prevalence in women beyond longevity alone. These biological, as well as social and lifestyle underpinnings contribute to differences in brain changes, progression, and symptom manifestation in AD between the genders (Mielke et al., 2014; Ferretti et al., 2018; Scheyer et al., 2018).

In fact, the longevity hypothesis does not take into account some important facts. First, the average life expectancy in the United States is currently 82 years for females and a little over 77 years for males, a difference of less than 5 years (Riedel et al., 2016). As male survival rates have been steadily increasing, studies in Europe anticipate the longevity gap to be less than 2 years by 2030 (Bennett et al., 2015). Second, statistical models have shown that women exhibit a twofold higher incidence and lifetime AD risk even after accounting for gender-dependent mortality rates, age at death, and differences in lifespan (Vina and Lloret, 2010; Carter et al., 2012).

Further, there are well-documented differences in brain anatomy, function, and age-related brain changes between men and women (Carter et al., 2012). Recent studies found that women tend to accumulate greater tangle burden than do men with the same brain Aβ levels, but with no difference in lifetime AD risk (Buckley et al., 2018), suggesting an earlier onset of AD pathophysiology. These observations are consistent with brain imaging findings of earlier emergence of AD-related brain changes in middle-aged women compared to age-matched men (Mosconi et al., 2017b, 2018). Women also exhibit greater rates of neuropathological decline after an AD diagnosis, as evidenced by increased hippocampal atrophy and neurofibrillary tangles compared to men (Barnes et al., 2005). In keeping with this, while women score generally higher on cognitive performance tests than men (Rentz et al., 2017), female AD patients exhibit a faster rate of cognitive decline and loss of independence in comparison to male patients at the same level of dementia severity (Mielke et al., 2014). Collectively, these data suggest an earlier start of AD pathogenesis in women, which might be masked by the female advantage in cognitive performance, resulting in females being diagnosed at a later stage than their male counterparts. Additionally, it highlights the importance of considering gender specific cut-offs in neuropsychological measures designed to detect AD-related cognitive impairments. Sex-adjusted cutoffs in the interpretation of verbal memory test results have led to improved diagnostic accuracy for both women and men (Sundermann et al., 2017a).

Furthermore, female sex may be associated with AD pathology seen in other conditions, like dementia with Lewy bodies, in which AD pathology occurs in a subset of patients. In one large study, while a composite AD biomarker profile was detected in 25% of all subjects, it was more frequent in women and was associated with worse cognitive performance (Van Steenoven et al., 2016). Given all these differences, further work into understanding sex differences in AD is an important step toward gender-based disease prevention.

## Genetic Risk Factors

### APOE Genotype

The APOE gene is currently the strongest genetic risk factor for late-onset AD (Harold et al., 2009). APOE codes for the Apolipoprotein E protein, an important cholesterol carrier that primarily coordinates transport of lipids in the brain. It consists of three major alleles: ε2, ε3, and ε4. APOE isoforms coordinate Aβ accumulation and removal in the brain, and play distinct roles in glucose metabolism, neuronal signaling, neuro inflammation, and mitochondrial function (Liu et al., 2013).

Individuals with the ε4 allele are at a higher AD risk compared to those with the more common ε3 allele, whereas the ε2 allele has been associated with decreased risk (Farrer et al., 1997). The ε4 allele is also associated with an earlier age onset in a gene dose-dependent manner (Corder et al., 1993). The frequency of AD and mean age at clinical onset for the different isoforms are as follows: 91% and 68 years of age in ε4 homozygotes, 47% and 76 years of age in ε4 heterozygotes, and 20% and 84 years in ε4 non-carriers (Corder et al., 1993). The ε4 allele is also related to an increased risk for cerebral amyloid angiopathy and age-related cognitive decline during normal aging (Liu et al., 2013).

Sex differences in the effects of the ε4 allele have been well documented, with female carriers being more likely than male carriers to develop AD (Farrer et al., 1997; Kim et al., 2009; Altmann et al., 2014; Ungar et al., 2014). AD risk increases nearly 4- and 10-fold in women with one and two ε4 alleles, whereas men exhibit essentially no increased risk with one ε4 allele and a fourfold increased risk with two ε4 alleles (Farrer et al., 1997; Kim et al., 2009). A recent longitudinal study demonstrated that the conversion risk from normal aging to MCI or AD and from MCI to AD conferred by the ε4 allele is also significantly greater in women compared to men (Altmann et al., 2014). However, a recent meta-analysis examining the relationship between APOE genotype and AD-dementia risk between men and women found no significant sex differences, except for a slightly increased risk for ε3/ε4 female carriers compared to male carriers within the ages of 65 and 75 (Neu et al., 2017).

Clearer evidence for negative associations of APOE ε4 genotype with female sex comes from biomarker studies showing that, among MCI patients, female ε4 carriers had significantly greater levels of CSF tau protein than male ε4 carriers (Altmann et al., 2014; Hohman et al., 2018). Among dementia-free individuals, female carriers exhibited greater brain hypometabolism, hippocampal volume reduction, and cortical thinning compared to male carriers (Altmann et al., 2014; Sampedro et al., 2015) Even in the absence of dementia, APOE ε4 significantly increases brain Aβ deposition and atrophy, and decreases brain connectivity in the default mode network much more effectively in women than in men (Fleisher et al., 2005; Damoiseaux et al., 2012; Mosconi et al., 2017b).

Given these findings, a greater comprehension of the APOE ε4 allele’s interaction with sex can have potential implications for AD treatment. To date, the few studies examining this issue have provided conflicting information (Berkowitz et al., 2018). A research study investigating the efficacy of Tacrine, an FDA-approved cholinesterase inhibitor for AD treatment, found that female ε2/ε3 carriers showed greater improvements compared to female ε4 carriers (Farlow et al., 1998). In contrast, men did not differ in their treatment responses based on APOE genotype (Farlow et al., 1998). Another study assessing the efficacy of anticholinesterase therapy showed that female ε4 carriers derived the greatest cognitive benefit compared to non-carriers (Macgowan et al., 1998). A study examining the efficacy of intranasal insulin on cognitive function found that ε4 negative males demonstrated improvements but female non-carriers did not derive any benefits (Claxton et al., 2013). Recent clinical trials of Aβ immunotherapy demonstrate that treatment was more effective in individuals with the ε4 genotype compared to non-carriers (Salloway et al., 2014), though the data were not broken down by sex. More work is needed to systematically examine the differential response to pharmacological interventions by sex and APOE genotype.

It is unclear why the APOE gene confers different risk in women, but some research suggests that it could be due to its interaction with estrogen (Yaffe et al., 2000; Kang and Grodstein, 2012). Studies in mice exhibited that APOE expression in different brain regions varied with the female reproductive cycle stages (Struble et al., 2003), consistent with the hypothesis that estradiol might induce APOE expression in the brain, as had already been demonstrated for APOE in blood (Srivastava et al., 1997). Moreover, trophic effects of estradiol on neurite growth in cultured mouse cerebral cortical neurons are reported to be highly dependent on APOE expression (Horsburgh et al., 2002).

Responses to estradiol are also in part dependent on APOE status: whereas estradiol is neurotrophic in the presence of human APOE ε2 or ε3, the ε4 variant does not support this response (Nathan et al., 2004). In keeping with these findings, several lines of evidence indicate differential effects of estrogen replacement therapy dependent on APOE status, with ε4 positive women exhibiting worse rates of cognitive decline compared to non-carriers (Yaffe et al., 2000; Kang and Grodstein, 2012). Interestingly, a recent study found that transdermal estrogen therapy was associated with reduced Aβ deposition in postmenopausal women, particularly in ε4 carriers (Kantarci et al., 2016). In contrast, oral doses of conjugated equine estrogens (CEEs) was not associated with lower Aβ deposition. These results highlight the interaction of the APOE ε4 allele with estrogen and provide support for a biologically medicated relationship between APOE, estrogen use, and cognitive impairments.

### Race

In general, older Hispanics and African Americans are at a higher AD risk in comparison to older whites (Alzheimer’s Association, 2017). Differences in various health, lifestyle, and socioeconomic factors likely contribute to their higher AD risk (Alzheimer’s Association, 2017). These include a greater prevalence of CVD, T2DM, hypertension, and early life adversity (Lines et al., 2014), as well as lower rates of education and physical activity (Glymour and Manly, 2008).

African American women in particular are twice more likely as white women to develop AD, strokes, and other forms of dementia (Alzheimer’s Association, 2002). Likewise, women of Hispanic origin have a one and a half times greater risk for developing dementia, as well as CVD and T2DM than those who are white (Alzheimer’s Association, 2017). This is of particular concern because in addition to a rapidly growing aging population, the United States is also becoming increasingly diverse. African Americans currently comprise 14.6% of the United States population and it is estimated that, by the year 2060, Hispanics who are currently the largest minority group will comprise over 28% of the United States population.

Additionally, the caregiving burden among women within these communities is especially high (Nebel et al., 2018). For instance, in some studies, Hispanic and African-American caregivers were more depressed and reported worse physical health than their white counterparts (Napoles et al., 2010). While data on minority groups remain limited, there is an ongoing effort to produce high-quality data on large numbers of racial and ethnic minorities to better understand and treat possible AD-related risk factors.

## Medical Risk Factors

### Cardiovascular Disease

Cardiovascular disease, including coronary heart disease, stroke, atrial fibrillation, and heart failure, is the leading cause of death worldwide and a major risk factor for AD (Hall et al., 2013; de Bruijn and Ikram, 2014). The association between CVD and AD has been attributed to shared modifiable risk factors such as hypertension, obesity, diabetes mellitus, high cholesterol, and smoking (de Bruijn and Ikram, 2014). Several studies point to alterations in brain gray matter volume, increases in white matter lesions, and subcortical damage related to CVD as factors that could potentially increase AD-related neurodegeneration risk (Hajjar et al., 2011).

Historically, CVD has been viewed as a typically “male” disease. The Framingham Heart study found that CVD related mortality and morbidity was two times higher in men than in women aged 50 and younger (Kannel et al., 1976). However, even though CVD risk increases with age in both genders, it shows a steeper increase in risk in women after the age of 50 coinciding with the loss of estrogens occurring during and after menopause (Möller-Leimkühler, 2007). Furthermore, coronary artery disease (CAD) is more prevalent in young females who underwent oophorectomy compared to those with intact ovaries (Parker et al., 2009).

Several studies have documented the protective role of estrogen in CVD via its role in regulating LDL-cholesterol levels (Mendelsohn and Karas, 1999; Iorga et al., 2017; Lagranha et al., 2018). During menopause, both natural and surgically induced, women experience an increase in LDL cholesterol levels. After age 50, LDL levels tend to increase at an average rate of 0.05 mmol/L per year in women aged 40–60 whereas they generally plateau in men (Johnson et al., 1993). This postmenopause induced increase in LDL levels could be explained by declining estradiol levels that result in a downregulation of the activity of LDL receptors in the liver. This, in turn, leads to a reduction in the clearance of LDL from blood serum levels (Pilote et al., 2007). Furthermore, estradiol’s interaction with ERα, ERβ, and GPER present in adult cardiomyocytes (Grohé et al., 1997; Ropero et al., 2006) exerts a protective role by increasing angiogenesis (new blood vessels formation from older vessels), improving mitochondrial activity and reducing oxidative stress and fibrosis (Iorga et al., 2017).

Sex differences in terms of CVD risk and underlying pathology have also begun to emerge.

Hypertension, a major risk factor for cognitive decline and a leading cause of cardiovascular morbidity, also increases significantly in women after menopause (Blacher et al., 2019). A meta-analysis found that for every 10 mmHg increase in systolic blood pressure, there was a 25% and 15% increase in CVD risk for women and men, respectively (Wei et al., 2017). Sex differences in terms of CVD treatment have also been documented. For example, statins may be less effective at lowering cholesterol in women compared to men (Assmann et al., 2006; Santos et al., 2009), although the complex relationship between statin exposure and sex-dependent risk reduction is complex and still remains to be understood (Zissimopoulos et al., 2017). Additionally, some clinical trials found that angiotensin receptor blockers improve survival rates in men, but not in women with hypertension or CVD (Fletcher et al., 1988; Rabi et al., 2008). The renin–angiotensin system is no an intense focus of research, given its potential association with risk of Alzheimer’s (Kehoe, 2018) and interaction of estrogen with this system (O’Hagan et al., 2012). Overall, hypertension seems to develop differently in women and men, and to respond differently to medications. The new guidelines by the American Heart Association for hypertension treatment will hopefully lead to better management of this risk factor in the future (Brook and Rajagopalan, 2018).

Stroke has also been associated with an increased AD risk and earlier age of onset for dementia (Honig et al., 2003). Sex differences in terms of the underlying causes of stroke have been documented. The two major types of strokes are ischemic (caused by a blood clot that blocks a vessel in the brain) and hemorrhagic (caused either by a brain aneurysm burst or a weakened blood vessel leak). Hemorrhagic stroke is the lesser common of the two but often results in death. Aneurysmal subarachnoid hemorrhage (aSAH) is higher in women than in men (De Marchis et al., 2017), possibly as the result of female specific factors such as repeated childbirths and hormonal changes. Pregnancy-induced hypertension and vascular tension during delivery may lead to the formation of aneurysms. Several studies have shown that the increased aSAH prevalence in women occurs after the age of 50, coinciding with postmenopausal-related estrogen declines (Kongable et al., 1996; Hamdan et al., 2014). However, a systematic review found that the role of hormone replacement therapy on the manifestation of aSAH is currently unclear (Feigin et al., 2005).

Finally, although sex differences in CAD have not been investigated adequately, there is some research indicating that women may be more prone to cardiac ischemia due to coronary microvascular obstruction than men (Jones et al., 2012). Women are also more affected by microvascular endothelial inflammation, a condition that contributes to heart failure (Jones et al., 2012). Compared to men, women who have experienced a myocardial infarction have a higher death rate, particularly evident in postmenopausal women, and experience more complications post-MRI such as stroke, congestive heart failure, cardiogenic shock, and depression (Shirato and Swan, 2010).

### Diabetes

Diabetes mellitus, a common condition characterized by dysregulation of insulin and glucose levels, increases risk for incident AD, MCI, and cognitive impairment (Biessels et al., 2006; Li et al., 2016) that posits a greater risk in women than men (Den Ruijter et al., 2015). For instance, women with type 1 diabetes mellitus (T1DM) exhibit a two times higher risk of cardiovascular events compared to men with T1DM (Huxley et al., 2015). This increased CVD risk has been associated with significantly worse cardiac risk profiles, poorer diabetes management, and treatment options in women (Humphries et al., 2017).

Type 2 diabetes mellitus is also linked to an increased CVD (Juutilainen et al., 2004) and AD risk in women, especially after menopause. The prevalence of T2DM increases with age in a sex-specific manner (Wild et al., 2004). The Study of Women’s Health Across the Nation (SWAN) found that declining estrogen levels resulted in a 47% greater T2DM risk during the MT (Park et al., 2017). The length of the reproductive lifetime, defined by age at last period and at menarche, has also been linked to women’s increased T2DM risk. The Women’s Health Initiative (WHI) showed that women with a reproductive lifetime of less than 30 years exhibited a nearly 40% increased T2DM risk than women with a lifetime reproductive span of 36–40 years (LeBlanc et al., 2017).

This menopausal-related increase in T2DM risk could be explained by biochemical and metabolic changes that take place during the MT (Slopien et al., 2018). For instance, it is linked to an increase in fat deposition (especially in the abdominal region), reduction in lean body mass, and decline in overall energy expenditure (Lovejoy et al., 2008; Leeners et al., 2017). The increased visceral fat accumulation leads to the development of insulin resistance (IR) and the MetS, which play a major role in the development of T2DM (Westphal, 2008). This finding is in accordance with previous data from experimental studies showing that reduced estrogen levels and decreased ERα activity is associated with IR development (Bryzgalova et al., 2006; Riant et al., 2009). Furthermore, T2DM and IR have been associated with atrophy of medial temporal regions such as the hippocampus and amygdala, which are particularly rich in ERs (den Heijer et al., 2003; Convit, 2005; Brinton et al., 2015). These results provide further support to T2DM as a risk factor for AD via dysfunction of insulin signaling.

### Depression

Depression falls among the most common mental disorders in the elderly and is strongly linked to a higher risk for cognitive decline in both genders (Yaffe et al., 1999; Wilson et al., 2002; Barnes et al., 2006; Verdelho et al., 2013). However, women are two times more likely than men to experience depression (Albert, 2015). Studies have shown a rapid increase in depression rates starting at puberty and continuing through adulthood in women (Piccinelli and Wilkinson, 2000). Vulnerabilities to mood disorders in women tend to coincide with hormonal fluctuations experienced during and after pregnancy, as well as at the MT, suggesting a link between sex hormones and depression (Steiner et al., 2003). For instance, women undergoing the MT experience a two- to threefold increase in major depressive disorder rates (Gordon et al., 2015). It has been well documented that during the perimenopause period, women are two to three times more likely than men to experience a first episode of depression (Nemeroff, 2007).

The association among sex, depression, and AD risk needs to be more carefully considered. The data in terms of depressive symptoms and cognition stratified by sex have been mixed. Some studies demonstrate a stronger inverse relationship among depression and cognitive function in women, whereas other studies exhibit a stronger association in men (Sundermann et al., 2017b). Furthermore, men with mild depressive symptoms exhibit an increased risk of amnestic MCI, while women with moderate or severe symptoms exhibit a higher AD risk (Sundermann et al., 2017b). This suggests that symptoms might have to meet a higher severity threshold to increase clinical risk conversion in women compared to men.

### Traumatic Brain Injury

Several studies suggest a link between traumatic brain injury (TBI) and an increased AD risk (Mortimer et al., 1991; Fleminger et al., 2003). Emerging evidence indicates that even mild TBI is linked to cortical thinning in AD-sensitive areas and reduced memory performance in patients at risk for AD (Hayes et al., 2017). Moreover, a history of TBI has been associated with AD neuropathology as evidenced by increased accumulation of Aβ and tau protein in patients with a history of TBI (Uryu et al., 2007). TBI is also associated with chronic brain inflammation which has been shown to further accelerate AD disease progression (Perry et al., 2007; Podcasy and Epperson, 2016).

Some studies have highlighted sex-based differences in the context of recovery from TBI from sports-related head injuries. Female athletes are at a significantly higher risk of poorer outcomes, greater symptom severity, and lower recovery rate following mild TBI and concussions compared to their male counterparts (Broshek et al., 2005; Bazarian et al., 2010). A recent MRI study focused on soccer related heading impacts found a sex-based association between heading and brain microstructure (Rubin et al., 2018). In response to similar levels of heading, females had a fivefold greater volume of affected white matter than men, demonstrating a higher burden of microstructural consequences.

The neuroprotective effects of estrogen in the context of recovery from TBI have been demonstrated in preclinical studies (Brotfain et al., 2016). Estrogen administration pre- and post-TBI is associated with increased neuronal survival, significant reductions in apoptosis, and improvements in functional outcomes (Soustiel et al., 2005; Day et al., 2013; Naderi et al., 2015). Estrogen is believed to be neuroprotective by increasing blood flow to ischemic regions after brain injuries, promoting antioxidant activity, and boosting the activity of astrocytes and microglia which provide neurons with metabolic support and elevate the immune response, respectively (Brotfain et al., 2016). Data from human studies show that mild TBI can potentially damage the anterior pituitary gland (Kelly et al., 2006; Klose et al., 2007), which is responsible for producing FSH and LH. This reduction could significantly disrupt the production and circulation of endogenous estrogen levels (Davis et al., 2006). The decline of estrogen associated with menopause could potentially explain the poorer outcomes exhibited by females post-TBI compared to males.

### Infections and Chronic Inflammation

Systemic infections and related inflammation may potentially lead to a worsening of AD symptoms and increase the progression of AD-related neurodegeneration (Perry et al., 2007). A retrospective study found that the occurrence of two or more infections within a 4-year time period was linked to an almost twofold greater risk of developing AD in men and women (Dunn et al., 2005). Following infections and injury, there is a heightened response of microglia and macrophages that lead to an increased inflammatory response.

Emerging evidence suggests that chronic inflammation in the brain may be central to AD pathogenesis and that this may be triggered through Aβ accumulation (Wyss-Coray, 2006). Postmortem brains examination of people with AD show increased expression of inflammatory mediators and complement factors, clusters of activated microglia, and cytokines in and near Aβ plaques (Hashioka et al., 2008; Minett et al., 2016). Although there is limited evidence that inflammation is a possible cause of late-onset AD, research on mouse models suggests that activation of inflammatory pathways is potent drivers of the disease (Wyss-Coray and Mucke, 2002). For instance, specific receptors on microglia and monocyte/macrophages are involved in determining whether Aβ clearance is carried out through non-inflammatory phagocytosis or via pro-inflammatory cytokine generation (Heneka et al., 2015). Further, gene expression related to inflammation in brain is increased in aging, and this effect is heightened in patients with AD (Villegas-Llerena et al., 2016). Some epidemiological studies also link anti-inflammatory drugs usage with reduced risk for the disorder, although results are not always consistent (Wyss-Coray, 2006).

Sex differences in terms of response and prevalence to infections and inflammation have been documented, with females experiencing greater disease severity and worse outcomes than males, especially in the presence of reduced estradiol levels (Klein et al., 2010). For instance, women are at a greater risk for chronic inflammatory conditions such as lupus, rheumatic arthritis, and multiple sclerosis, especially after menopause (Straub and Schradin, 2016). Additionally, preclinical studies demonstrate that the presence of influenza infection was associated with reduced reproductive functions in females (Robinson et al., 2011). Furthermore, females treated with estradiol or an ERα receptor agonist had improved survival rates compared to females with either low levels or no estradiol (Robinson et al., 2011).

Overall, these findings suggest that sex differences in microglia activity in response to fluctuating hormone levels may lead to increased inflammatory responses, which may in turn increase women’s vulnerability to AD related neurodegeneration in later life stages (Peterson et al., 2015; Hanamsagar and Bilbo, 2016).

## Hormonal Risk Factors

### Thyroid Disease

Thyroid function is routinely screened for in the clinical assessment of AD because thyroid dysfunction can cause symptoms that mimic those of dementia (Tan and Vasan, 2009). Thyroid complications arise from an imbalance of triiodothyronine (T3) and thyroxine (T4) hormones, which regulate metabolism and vital functions. Hypothyroidism and hyperthyroidism result from an under and over production of T3 and T4 hormones, respectively. Among other potential causes, Graves’ disease and Hashimoto’s disease (two autoimmune conditions) are the most common causes of hyper- and hypothyroidism.

It is widely reported that women are more likely to experience thyroid problems than men (del Ghianda et al., 2014). One in eight women is expected to be affected by thyroid problems throughout their lifetimes. Some evidence shows that thyroid hormones can interfere with menstrual cycles and cause problems during pregnancy (discussed below) by reducing the clearance of estradiol and acting synergistically with FSH to increase the production of progesterone (Yen, 1986; Cecconi et al., 1999).

### Pregnancy

Pregnancy and childbirth are characterized by obvious fluctuations in hormonal regulation that causes wide-ranged metabolic changes. Sometimes these can lead to a higher occurrence of IR and dyslipidemia, with a greater risk of future diabetes and obesity, all of which could potentially exacerbate AD risk later in life (Cohen et al., 2006). There are mixed results on whether pregnancy increases AD risk later in life. Some studies report that a higher number of pregnancies are indeed linked to a higher risk and an earlier age of AD onset (Sobów and Kloszewska, 2003; Colucci et al., 2006). For instance, one study estimated that women who had at least three pregnancies had a threefold greater risk of developing AD (Colucci et al., 2006). The number of children born is also linked to increased neuropathological lesions of AD in women (Beeri et al., 2009). However, a recent study reported the opposite trend, with a higher number of pregnancies linked to a lower AD risk in later life (Fox et al., 2018).

Even though the data on pregnancy have been mixed, pregnancy-related conditions such as gestational diabetes and preeclampsia (pregnancy-related hypertension) can worsen CVD risk, and therefore risk of dementia (Garovic et al., 2010). Further, hypertension due to pregnancy and vascular tension during delivery can potentially lead to aneurysms formation, which can contribute to an increased risk of stroke later in life.

### Menopause

**FIGURE 2:**
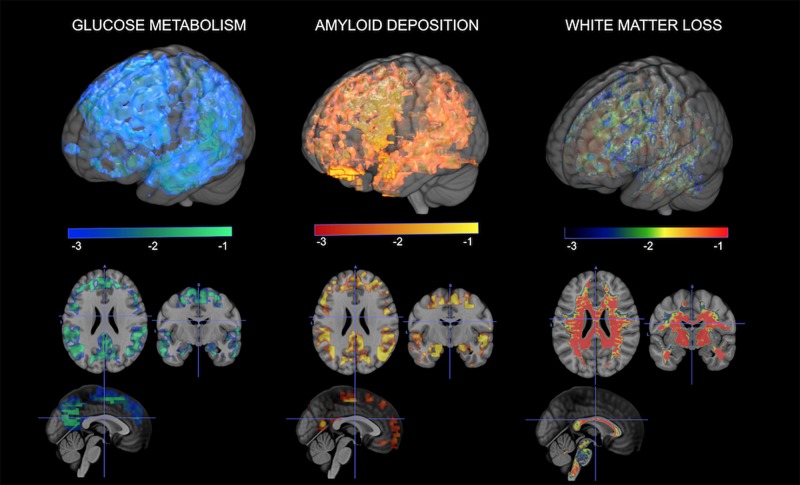
Multi-modality brain imaging of the menopausal transition. From left to right: 3D statistical parametric maps (SPMs) depicting areas of brain hypometabolism, increased amyloid-beta deposition, and white matter loss in peri- and postmenopausal women relative to age-matched men. Corresponding *Z* scores are displayed using a color coded scale at *p* < 0.001.

As mentioned throughout the article, the MT is the only known female-specific risk factor for AD to date (Brinton et al., 2015). The effects of MT on AD risk have been highlighted by neuroimaging studies demonstrating a link between menopausal changes and emergence of AD pathology in midlife (Mosconi et al., 2017a, 2018; Scheyer et al., 2018; Figure 2). Among cognitively intact participants, postmenopausal and perimenopausal women exhibit higher AD-burden, as reflected by reduced glucose metabolism, increased Aβ deposition, gray matter volume loss (atrophy), and white matter volume loss than premenopausal women and age-matched men (Mosconi et al., 2017b). Furthermore, a 3-year longitudinal study demonstrated that postmenopausal and perimenopausal women exhibited higher rates of AD biomarker progression, as evidenced by greater rates of metabolic declines and Aβ accumulation (Mosconi et al., 2018). These data point to the MT overlapping with the time course of preclinical AD. This is also supported by studies showing that estrogen depletion following oophorectomy is linked to an increased AD risk by up to 70% (Rocca et al., 2007, 2014; Phung et al., 2010).

Altogether, research provides support to the idea of the MT as an “optimal window of opportunity” for AD preventative interventions in women. The “critical window hypothesis,” also known as “the timing hypothesis” or “the critical period hypothesis,” says that the impact of hormonal replacement therapy (HRT) depends on the timing of treatment onset with respect to age and/or menopause onset, with benefits pertaining to early initiation (Maki, 2013).

This is in stark contrast with the historical blanket use of high-dose HRT for treatment of menopause symptoms in postmenopausal women, which was common practice from the 1960s through 2003. In 2003, the primary results from the WHI study, a pivotal study investigating the effect of HRT on women’s health, were published. The WHI had two arms, one for hysterectomized women where the active treatment was estrogen-alone therapy (ET), and the other for postmenopausal women with a uterus where the active treatment was estrogen-plus-progestin therapy (EPT). Both trials were interrupted as early results showed a higher risk of CAD, stroke, and blood clots, with the EPT arm of the study also showing an increased risk of cancer (Rossouw et al., 2002; Anderson et al., 2004).

Further, the WHI included an additional arm, the WHI Memory Study (WHIMS), which investigated the outcome of HRT on dementia risk (Shumaker et al., 2003). In order to test whether HRT was effective in dementia prevention, the trial focused on postmenopausal women who were aged 65 or older at the time of enrollment. From a public health standpoint, it was thought that those women had the most to gain from the intervention since they were the most vulnerable to developing AD, as well as other conditions like CVD that could further increase AD risk. In the EPT arm, with a sample size of 4,532 women, there was a doubling of the risk of all-cause dementia with active treatment compared to placebo after an average follow-up of 4 years (Shumaker et al., 2003). The ET arm, with a sample size of 2,947 hysterectomized women followed over an average of 5 years, reported no significant impact of ET on dementia risk (Marder and Sano, 2000). These findings were in striking contrast to previous observational studies reporting a reduced risk of AD among women who had used HRT compared to those who had not (Zandi et al., 2002) as well as with smaller clinical trials showing no effects of HRT in AD patients (Mulnard et al., 2000; Wang et al., 2000).

It is important to note several limitations pertaining to the WHI trials. First, the treatment administered was in the form of CEE tablets rather than 17 beta estradiol, with or without continuous medroxyprogesterone acetate, depending on the subject’s hysterectomy status (Rossouw et al., 2002). This might not have produced the same effects as oral or transdermal administration of estrogen or progesterone. Additionally, participants were already postmenopausal, generally older than 65 at the time of enrollment (therefore several years into menopause), possibly with pre-existing cardiovascular conditions. This raises the question of whether the results are applicable to younger post or peri-menopausal women. More work is needed to better examine effects of HRT dose, formulation, and mode of delivery on women’s brain health, especially for younger women without pre-existing conditions.

Recent re-examination of results from the WHIMS indicates that treatment risks and benefits associated with HRT largely depend on three main factors: the patient’s chronological age, endocrine age (years to/from menopause), and hysterectomy status. Re-examination of the WHI data 18 years after they were interrupted reported that women who initiated HRT before the age of 60 or within 10 years after menopause had a lower mortality rate than placebo (Salpeter et al., 2004; Manson et al., 2017). Further, the Early versus Late Intervention Trial with Estradiol (ELITE) conducted with more than 600 postmenopausal women provided evidence that HRT reduced the progression of subclinical atherosclerosis when therapy was initiated right after menopause onset (Hodis et al., 2016), which has been associated with a 30% reduced number of heart attacks and cardiac deaths (Salpeter et al., 2009).

It is possible that early initiation of estrogen therapy may also provide protection against dementia later in life. Results on this topic have been mixed. On the one hand, meta-analysis of 18 studies demonstrated that among younger, 50–59-year-old women, those who used HRT had a 30–44% reduction in AD risk compared to those who did not use HRT (LeBlanc et al., 2001; Maki, 2013), although these data need to be verified in formal clinical trials. In contrast, two recent randomized clinical trials – the ELITE study mentioned above and the Kronos Early Estrogen Prevention Study (KEEPS) – showed no cognitive improvements in women who started HRT within 6 years of menopause, but also no adverse effects of HRT (Gleason et al., 2015; Henderson et al., 2016; Miller et al., 2019). As both trials focused on women who were several years past menopause, more work is needed to systematically look at HRT effects in younger women, especially those of perimenopausal age.

More persuasive evidence that HRT has value for dementia prevention comes from studies of hysterectomized women, particularly those who had their ovaries removed (Rocca et al., 2007). A recent epidemiological study of 1,884 women showed that those who initiated ET within 5 years of surgery and continued until the natural age at menopause had a lower AD risk compared to those who did not take the drug (Bove et al., 2014). Additionally, randomized clinical trials of younger hysterectomized women showed that ET therapy had general beneficial effects on memory performance (Maki, 2013).

Taken together, the majority of studies suggest that, for women with a uterus, EPT therapy initiated within 5 years of menopause onset or in the perimenopausal period may lower AD risk, whereas initiating therapy more than 5 years postmenopause may have the opposite effect. For women without a uterus, ET therapy started as close as possible after surgery and continuing until the natural age of menopause may offset the negative effects of the surgeries and also reduce AD risk (Rocca et al., 2012). The value of initiating ET after the natural age at menopause is unclear.

## Lifestyle Factors

As previously discussed by Nebel et al. (2018), in medical research, the term “sex” refers to biological differences such as chromosomal or hormonal factors, whereas “gender” refers to differences in the impact of psychosocial, cultural, and environmental influences on biological factors between men and women. Gender-related risk factors for AD are discussed below.

### Educational Attainment, Occupation, and Intellectual Activity

Low levels of educational achievement and occupation are associated with an increased AD risk in both genders (Katzman, 1993; Stern et al., 1994; Karp et al., 2004). A possible explanation for this relationship lies in the idea of “cognitive reserve,” the brain’s ability to effectively utilize cognitive networks to allow individuals to normally perform cognitive activities despite sustaining pathological brain abnormalities such as increases in Aβ and tau levels (Stern, 2012). Higher education levels and cognitively stimulating occupations build more cognitive reserve. Likewise, several systematic reviews demonstrate that participation in cognitively stimulating activities is linked to a lower dementia risk (Stern and Munn, 2010; Wang et al., 2012; Fallahpour et al., 2016).

Historically, women had limited access to educational opportunities compared to men, which may have put them at a disadvantage in terms of their cognitive reserve build-up. This is especially relevant for people currently aged 70 years or older, who are at the greatest risk for AD. These findings suggest that lower educational achievement in women compared to men born early on in the 20th century could potentially play a role in women’s increased AD prevalence. However, according to the recent census, women had a higher educational attainment in the United States compared to men (Ryan and Siebens, 2012). There has been a significant change in occupational engagement with women taking on higher level positions and other roles that used to be men’s prerogatives. Hopefully these changing trends may help reduce the prevalence of Alzheimer’s in women in the future.

Although studies that examined gender differences between cognitively engaging tasks and dementia risk are scarce, a recent study demonstrated that higher engagement in intellectual-cultural activities such as reading, radio and TV, and partaking in social and cultural activities were associated with improved verbal abilities in women, whereas higher engagement in self-improvement activities (playing sports, clubs and organizations, studies, outdoor activities) were associated with improved cognitive function in men (Hassing, 2017). Despite the fact that women generally partake in more cognitive activities such as reading, arts and crafts, and social activities, the effect of these activities on cognitive reserve may be weaker than that of educational and occupational attainment (Mielke et al., 2014; Vemuri and Lesnick, in press).

### Physical Activity

Low levels of physical activity are associated with a higher risk of dementia and greater cognitive decline among older adults (Groot et al., 2016; Tan et al., 2016; Willey et al., 2016). Physical activity can improve cognition through indirect effects on modifiable risk factors such as hypertension, obesity, IR, or via direct effects on neuronal activity (Van Praag, 2009; Livingston et al., 2017). Increased physical activity has been shown to promote the formation, survival, and synaptic plasticity of new neurons in the hippocampus (Van Praag et al., 1999; Farmer et al., 2004; Van Praag, 2008), and increase the production of brain-derived neurotrophic factor (BDNF) which play an important role in the formation, growth, and plasticity of neurons (Mulnard et al., 2000).

Neuroimaging studies have also shown the beneficial effects of physical activity on brain structure and function (Hillman et al., 2008). Individuals in a 3-month fitness training program showed increases in blood flow to the hippocampus, which was linked to improvements on memory and verbal learning tasks (Pereira et al., 2007). Cross-sectional MRI studies report that increased fitness activity was related to larger anterior matter, prefrontal and temporal gray matter volumes (Colcombe et al., 2004, 2006; Marks et al., 2007; Gordon et al., 2008). An fMRI study showed that individuals who underwent an aerobic fitness training program exhibited increased activity in the middle frontal gyrus, superior parietal cortex, and significant improvements in cognitive performance (Colcombe et al., 2004).

Despite the well-known link between exercise and improved brain function, a gender gap exists in terms of physical activity engagement. For instance, women tend to engage in less physical activities than men (Troiano et al., 2008; Edwards and Sackett, 2016). Research suggests that women’s societal roles can play a role, as parenthood and marital status may hinder women’s participation in physical activities (Verhoef et al., 1993). The prevalence of higher physical inactivity among women is concerning due to its association with T2DM, CVD, obesity, and hypertension (Barnes et al., 2005).

However, the relationship between long-term exercise and reduced cognitive impairment and AD dementia risk is more pronounced in women than men (Laurin et al., 2001). For example, a meta-analysis examining the relationship between fitness training levels and cognition in older adults showed that fitness-related benefits on cognition were greater in women compared to men (Colcombe and Kramer, 2003). A study conducted with over 9,000 women found that, although exercising in the teenage years was particularly brain-protective in the long term, being physically active reduced risk of cognitive impairment no matter their age (Middleton et al., 2010). Elderly women with greater physical activity exhibit a reduced risk for AD dementia and are less likely to experience cognitive decline compared to women with lower physical activity levels (Yaffe et al., 2001). Importantly, exercise has been shown to ameliorate cognitive deficits even in women with a diagnosis of cognitive impairment and dementia (Eggermont et al., 2006; Hogervorst et al., 2012).

### Diet

Specific dietary patterns like the Mediterranean (MeDi) diet and the Mediterranean-DASH Intervention for Neurodegenerative Delay (MIND) diet have been associated with a reduced risk of dementia in both genders (Scarmeas et al., 2006, 2018; Mosconi et al., 2014; Morris et al., 2015). Further, higher MeDi adherence is associated with a lower risk of AD biomarker abnormalities such as hypometabolism and Aβ deposition in AD-sensitive brain regions already in midlife (Mosconi et al., 2014; Berti et al., 2018).

Both these dietary patterns focus on consumption of vegetables, fruit, whole grains, and legumes, with moderate amounts of fish and poultry, and limited amounts of dairy, red meat, and alcohol. Although results are not always consistent, several observational studies have shown that a MeDi-style diet is particularly protective for women, conferring a lower risk of AD, CVD, and diabetes (Gu et al., 2010; Gu and Scarmeas, 2011; Kaczmarczyk et al., 2012). A study conducted exclusively in elderly women showed that higher MeDi adherence was also moderately related to better cognition and verbal memory (Samieri et al., 2013; Berendsen et al., 2018). A recent study investigating the effect of a coconut oil enriched MeDi diet in mild to moderate AD patients found that women derived greater cognitive benefits than men (de la Rubia Ortí et al., 2018).

Higher whole grains and legumes consumption have also been related to reduced CVD risk, T2DM, obesity, and MetS in women (Rietjens et al., 2017) which could be attributed to the fact that they contain high concentrations of phytoestrogens. Phytoestrogens are polyphenols which have similar molecular structure to endogenous estrogen. Evidence suggests that phytoestrogens exert their beneficial effects on female physiology via binding to ERs, activating epigenetic mechanisms, and increasing antioxidant activity (Kuiper et al., 1998; Casanova et al., 1999; Jungbauer and Medjakovic, 2014; Remely et al., 2015). Furthermore, some preclinical studies have shown beneficial effects of phytoestrogens on cognitive function and AD (Um et al., 2009; Giridharan et al., 2011; Hu et al., 2012; Jeong et al., 2013). Perhaps as a result, increased intake of legumes and fish has been associated with a delayed menopause onset, whereas refined carbs, sugar, and processed foods resulted in an earlier onset of menopause and reduced ovulatory fertility (Nagel et al., 2005).

Other nutrients have also been associated with improved health outcomes in women, especially those past menopause. The Nurses’ Health Study conducted on over 75,000 women showed that replacement of saturated and trans-unsaturated fat for carbohydrates significantly reduced the risk of heart attack and stroke later in life (Hu et al., 1997). For each 5% energy intake increase from saturated fat, compared to the same energy intake from carbohydrates, a 17% increase in coronary disease risk was observed. Many other studies have shown that diets that favor carbohydrates, especially those with low glycemic load and high fiber content, also reduce T2DM risk (Liu et al., 2000; Schulze et al., 2004) and breast cancer in women (Monroe et al., 2007). Further, replacement of 5% of energy from saturated fat with energy from unsaturated fats was estimated to reduce CVD risk in women by 42%, while replacement of 2% of energy from trans-fat with energy from un-hydrogenated, unsaturated fats would reduce risk by 53% (Hu et al., 1997). These data suggest that a similar nutritional pattern might be protective against AD in women as well, given that higher intakes of saturated fat and trans-fat have been linked to an almost doubled risk of dementia, whereas higher intake of unsaturated fat has been linked to a reduced risk (Morris et al., 2004; Okereke et al., 2012; Morris and Tangney, 2014).

### Sleep

Poor sleep quality and circadian rhythm disruptions have been associated with an increased AD risk in the elderly (Ju et al., 2013, 2014; Spira et al., 2014). Preclinical and human studies have confirmed the beneficial effects of sleep via increases in cerebral blood flow and the clearing of Aβ plaques by microglial cells and astrocytes (Mangold et al., 1955; Xie et al., 2013). Sleep deprivation leads to an increase in Aβ plaques accumulation (Shokri-Kojori et al., 2018). Subjective measures such as self-reports of sleep deterioration have also been linked to greater Aβ burden (Sprecher et al., 2015).

When compared to men, women are generally at a greater risk for insomnia and experience greater age-dependent sleep quality deterioration, especially after menopause (Madrid-Valero et al., 2017; Auer et al., 2018). Sleep apnea, a condition marked by recurring interruption of breathing during sleep, has been linked to cognitive decline and AD risk (Ancoli-Israel et al., 2008). Even though sleep apnea affects more men than women, its incidence significantly increases after menopause in women (Bixler et al., 1998). Declining levels of estrogen and progesterone are thought to contribute to these findings. The Nurses’ Health Study II showed that women who underwent surgical menopause, which results in a shorter lifetime exposure to estrogen, had a 26% higher risk of experiencing obstructive sleep apnea (OSA) (Huang et al., 2018). Even though the exact underlying biological mechanisms remain unclear, few studies have suggested that estrogen contributes to OSA risk by acting on upper airway dilatory pathways to coordinate ventilation (Popovic and White, 1998; Pillar et al., 2000). This sex-specific increase is concerning because women experiencing sleep disturbances are more prone to metabolic and cardiovascular dysfunction and mood disorders such as depression, previously established AD risk factors (Mong et al., 2011).

Historically, women have been widely underrepresented in sleep studies which means that our current understanding of sleep-related disorders mostly comes from research conducted in men (Mong et al., 2011). This imbalance has significant implications for the efficacy of treatment interventions since strategies catered to men might not be effective or applicable to women.

### Stress

Cortisol dysregulation associated with repeated activation of the hypothalamic pituitary adrenal (HPA) axis in response to chronic stress is commonly found in patients with AD (Giubilei et al., 2001). It has been linked to memory impairments, cognitive decline, as well as brain atrophy (Huang et al., 2009; Rothman and Mattson, 2010; Brureau et al., 2013).

Sex-related differences in the HPA axis reactivity to early childhood and chronic stress have been previously reported. In response to early childhood trauma, women exhibit a significantly lower cortisol response compared to men later on in life (DeSantis et al., 2011). Moreover, this blunted HPA axis response occurs in a dose-dependent manner. The gender differences with regard to chronic stress could potentially be explained by the activity of gonadal hormones (Stephens et al., 2016). An fMRI study demonstrated that brain circuitry activation patterns in response to stress in menis more similar to women in the early follicular phase of the menstrual cycle, during which estrogen and progesterone are low, compared to women in the late follicular/midcycle phase, during which estrogen is high and progesterone is still relatively low (Goldstein et al., 2010). Women demonstrated lower stress response circuitry activation compared to men, with differences being particularly evident as they progressed through their menstrual cycles. This finding implies that hormonal changes specifically estrogen or progesterone could potentially account for these activation differences. Additionally, it raises the question of whether stress impacts brain aging and neurodegeneration differently in women and men. A recent imaging study conducted in cognitively normal middle-aged adults demonstrated that increased cortisol levels were linked to brain volume reductions and impaired memory, with the brain shrinkage being only evident in women (Echouffo-Tcheugui et al., 2018), which further highlights the link between hormonal changes and stress reactivity in sex differences (Goldstein et al., 2010; Bale and Epperson, 2015).

### Caregiver Burden

Research indicates that caregiving demands can severely tax the caregivers’ health and physical abilities, while compromising their immune response to stress, a condition known as “caregiver burden.” Caregiver burden has been associated with increased stress, sleep disturbances, depression, difficulties in social functioning, and declines in cognitive function (Alzheimer’s Association, 2017). At the same time, the stress associated with caregiving can worsen existing chronic health conditions (Navaie-Waliser et al., 2002), with higher rates of cholesterol, blood pressure, and obesity (Anderson et al., 2010). This has been associated with a greater risk of heart disease, stroke, and premature mortality, particularly under conditions of high strain (Schulz and Beach, 1999). Moreover, perhaps due to all the reasons above, caregivers are at a greater risk for developing AD themselves (Dassel et al., 2017). Approximately two-thirds of caregivers for AD dementia are women (Alzheimer’s Association, 2017).

In keeping with the notion that women’s reaction to stress is stronger than men’s, female caregivers report twice as more caregiver burden than their male counterparts. A study examining biological and emotional responses among spousal caregivers of patients with AD found that men reported significantly lower levels of stress, depression, subjective caregiver burden, and anxiety than women (Thompson et al., 2004). Additionally, men reported higher levels of mental health functioning, sense of coherence, and social and physical well-being. The gender differences could be partly due to the fact that women tend to devote longer hours and perform a higher number of caregiving tasks than men (Pinquart and Sörensen, 2003).

### Marital Status

Longitudinal studies have demonstrated that unmarried or single individuals are at an increased risk for cognitive decline, MCI, and AD (Helmer et al., 1999; Sundström et al., 2014). Currently, marital status is the only AD risk factor that affects men more than women. While single people tend to have twice the risk of developing AD versus people with partners (Håkansson et al., 2009), non-cohabiting men are at a greater risk of experiencing cognitive impairment later in life compared with non-cohabitating women (Håkansson et al., 2009). Divorced men also exhibit a higher AD risk compared to divorced women (Sundström et al., 2014). Interestingly, this difference was reduced after adjusting for socioeconomic (e.g., education and income) and demographic characteristics (e.g., age) suggesting that these factors could reduce risk in both genders. For instance, it has been shown that widowed women have a greater tendency be more socially active compared to widowed men which might reduce the negative effects of widowhood (Dykstra and de Jong Gierveld, 2004).

## Conclusion

In conclusion, AD is a neurodegenerative disorder that has shown strong sex and gender differences in several aspects of the disease, including a faster onset of AD pathology and disease progression after diagnosis, and different risk factors that may account for the increased female prevalence of AD. This review article focused on the research dedicated to understanding the effects of estradiol in terms of gender and sex differences in AD, and the negative effects of MT as a tipping point for middle-aged women. The research findings presented range from studies on molecular mechanisms and preclinical models that clearly highlight estradiol’s interactions with a number of signaling and transcriptional pathways involved in cognition and memory, to neuroimaging studies that visualized AD-related brain changes during the MT. Recent clinical trials and re-examinations of existing data lend support to the use of HRT as a possible risk reduction intervention in women at risk for AD, though more work is needed to examine this. Future research studies examining the underlying mechanism of estradiol’s neuroprotective action in AD are warranted.

In order to address the growing AD epidemic, the field is shifting toward early detection and primary and secondary prevention efforts (Isaacson et al., 2018). It is crucial that these prevention and clinical trials take into account sex differences in AD biomarkers, disease progression, and gender differences with respect to modifiable risk factors to aid in the development of therapeutics for both men and women. Historically, women have been underrepresented in studies elucidating the underlying mechanisms of AD which has significantly impeded our understanding of gender differences (Mazure and Jones, 2015). Women still remain underrepresented in clinical trials of CVD, a known AD risk factor (Shen and Melloni, 2014). Future AD research studies should actively aim to increase women’s overall participation and analyze the influence of sex or gender on health outcomes. A better understanding of sex and gender differences is crucial toward the development of individualized AD risk reduction strategies and treatments.

## Author Contributions

LM and AR discussed the concepts and wrote the manuscript. HJ, HH, RI, NS, TS, OE, CH, and RB reviewed the literature and provided critical revision of the manuscript for important intellectual content.

## Conflict of Interest

The authors declare that the research was conducted in the absence of any commercial or financial relationships that could be construed as a potential conflict of interest.

## References

[B1] Albert P. R. (2015). Why is depression more prevalent in women? *J. Psychiatry Neurosci.* 40 219–221. 10.1503/jpn.150205 26107348 PMC4478054

[B2] Altmann A. Tian L. Henderson V. W. Greicius M. D. Investigators A. S. D. N. I. (2014). Sex modifies the APOE-related risk of developing Alzheimer disease. *Ann. Neurol.* 75 563–573. 10.1002/ana.24135 24623176 PMC4117990

[B3] Alzheimer’s Association (2002). *African Americans and Alzheimer’s disease: The Silent Epidemic.* Chicago, IL: Alzheimer’s Association.

[B4] Alzheimer’s Association (2017). 2017 Alzheimer’s disease facts and figures. *Alzheimers Dement.* 13 325–373. 10.1016/j.jalz.2017.02.001

[B5] Ancoli-Israel S. Palmer B. W. Cooke J. R. Corey-Bloom J. Fiorentino L. Natarajan L. (2008). Cognitive effects of treating obstructive sleep apnea in Alzheimer’s disease: a randomized controlled study. *J. Am. Geriatr. Soc.* 56 2076–2081. 10.1111/j.1532-5415.2008.01934.x 18795985 PMC2585146

[B6] Anderson G. L. Limacher M. C. Assaf A. R. Bassford T. Beresford S. A. Black H. R. (2004). Effects of conjugated equine estrogen in postmenopausal women with hysterectomy: the Women’s Health Initiative randomized controlled trial. *JAMA* 291 1701–1712. 15082697 10.1001/jama.291.14.1701

[B7] Anderson N. B. Nordal K. Breckler S. Ballard D. Bufka L. Bossolo L. (2010). Stress in America findings. *Am. Psychol. Assoc.* 42:60.

[B8] Andrieu S. Coley N. Lovestone S. Aisen P. S. Vellas B. (2015). Prevention of sporadic Alzheimer’s disease: lessons learned from clinical trials and future directions. *Lancet Neurol.* 14 926–944. 10.1016/s1474-4422(15)00153-2 26213339

[B9] Assmann G. Benecke H. Neiss A. Cullen P. Schulte H. Bestehorn K. (2006). Gap between guidelines and practice: attainment of treatment targets in patients with primary hypercholesterolemia starting statin therapy. Results of the 4E-Registry (Efficacy calculation and measurement of cardiovascular and cerebrovascular events including physicians’ experience and evaluation). *Eur. J. Cardiovasc. Prev. Rehabil.* 13 776–783. 10.1097/01.hjr.0000189805.76482.6e 17001218

[B10] Auer M. Frauscher B. Hochleitner M. Hoegl B. (2018). Gender-specific differences in access to polysomnography and prevalence of sleep disorders. *J. Womens Health* 27 525–530. 10.1089/jwh.2017.6482 29356591

[B11] Bacon E. R. Mishra A. Wang Y. Desai M. K. Yin F. Brinton R. D. (2019). Neuroendocrine aging precedes perimenopause and is regulated by DNA methylation. *Neurobiol. Aging* 74 213–224. 10.1016/j.neurobiolaging.2018.09.029 30497015 PMC7117064

[B12] Bale T. L. Epperson C. N. (2015). Sex differences and stress across the lifespan. *Nat. Neurosci.* 18 1413–1420. 10.1038/nn.4112 26404716 PMC4620712

[B13] Balthazart J. Ball G. F. (2006). Is brain estradiol a hormone or a neurotransmitter? *Trends Neurosci.* 29 241–249. 10.1016/j.tins.2006.03.004 16580076

[B14] Barnes D. E. Alexopoulos G. S. Lopez O. L. Williamson J. D. Yaffe K. (2006). Depressive symptoms, vascular disease, and mild cognitive impairment: findings from the Cardiovascular Health Study. *Arch. Gen. Psychiatry* 63 273–279. 16520432 10.1001/archpsyc.63.3.273

[B15] Barnes L. L. Wilson R. S. Bienias J. L. Schneider J. A. Evans D. A. Bennett D. A. (2005). Sex differences in the clinical manifestations of Alzheimer disease pathology. *Arch. Gen. Psychiatry* 62 685–691. 15939846 10.1001/archpsyc.62.6.685

[B16] Bazarian J. J. Blyth B. Mookerjee S. He H. McDermott M. P. (2010). Sex differences in outcome after mild traumatic brain injury. *J. Neurotrauma* 27 527–539. 10.1089/neu.2009.1068 19938945 PMC2867588

[B17] Beeri M. S. Rapp M. Schmeidler J. Reichenberg A. Purohit D. P. Perl D. P. (2009). Number of children is associated with neuropathology of Alzheimer’s disease in women. *Neurobiol. Aging* 30 1184–1191. 18079025 10.1016/j.neurobiolaging.2007.11.011 PMC2718710

[B18] Bennett J. E. Li G. Foreman K. Best N. Kontis V. Pearson C. (2015). The future of life expectancy and life expectancy inequalities in England and Wales: bayesian spatiotemporal forecasting. *Lancet* 386 163–170. 10.1016/s0140-6736(15)60296-3 25935825 PMC4502253

[B19] BerendsenA. M.KangJ.FeskensE.de GrootC.GrodsteinF.Van de RestO. (2018). Association of long-term adherence to the mind diet with cognitive function and cognitive decline in American women. *J. Nutr. Health Aging* 22 222–229. 10.1007/s12603-017-0909-0 29380849

[B20] Berkowitz C. Mosconi L. Rahman A. Scheyer O. Hristov H. Isaacson R. S. (2018). Clinical application of APOE in alzheimer’s prevention: a precision medicine approach. *J. Prev. Alzheimers Dis.* 5 245–252. 30298183 10.14283/jpad.2018.35 PMC6188641

[B21] Berti V. Walters M. Sterling J. Quinn C. G. Logue M. Andrews R. (2018). Mediterranean diet and 3-year Alzheimer brain biomarker changes in middle-aged adults. *Neurology* 90 e1789–e1798. 10.1212/wnl.0000000000005527 29653991 PMC5957301

[B22] Biessels G. J. Staekenborg S. Brunner E. Brayne C. Scheltens P. (2006). Risk of dementia in diabetes mellitus: a systematic review. *Lancet Neurol.* 5 64–74. 10.1016/s1474-4422(05)70284-2 16361024

[B23] Bixler E. O. Vgontzas A. N. Ten Have T. Tyson K. Kales A. (1998). Effects of age on sleep apnea in men: I. Prevalence and severity. *Am. J. Respir. Crit. Care Med.* 157 144–148. 10.1164/ajrccm.157.1.9706079 9445292

[B24] Blacher J. Kretz S. Sorbets E. Lelong H. Vallée A. Lopez-Sublet M. (2019). Epidemiology of hypertension: differences between women and men. *Presse Med.* 10.1016/j.lpm.2019.04.010 [Epub ahead of print]. 31151845

[B25] Bove R. Secor E. Chibnik L. B. Barnes L. L. Schneider J. A. Bennett D. A. (2014). Age at surgical menopause influences cognitive decline and Alzheimer pathology in older women. *Neurology* 82 222–229. 10.1212/wnl.0000000000000033 24336141 PMC3902759

[B26] Brinton R. D. (2004). Impact of estrogen therapy on Alzheimer’s disease. *CNS Drugs* 18 405–422. 10.2165/00023210-200418070-00001 15139797

[B27] Brinton R. D. (2008). The healthy cell bias of estrogen action: mitochondrial bioenergetics and neurological implications. *Trends Neurosci.* 31 529–537. 10.1016/j.tins.2008.07.003 18774188 PMC10124615

[B28] Brinton R. D. (2009). Estrogen-induced plasticity from cells to circuits: predictions for cognitive function. *Trends Pharmacol. Sci.* 30 212–222. 10.1016/j.tips.2008.12.006 19299024 PMC3167490

[B29] Brinton R. D. (2017). *Reproductive Aging of Neuroendocrine Systems.* Tucson, AZ: University of Arizona.

[B30] Brinton R. D. Yao J. Yin F. Mack W. J. Cadenas E. (2015). Perimenopause as a neurological transition state. *Nat. Rev. Endocrinol.* 11 393–405. 10.1038/nrendo.2015.82 26007613 PMC9934205

[B31] Brook R. D. Rajagopalan S. (2018). 2017 ACC/AHA/AAPA/ABC/ACPM/AGS/APhA/ASH/ASPC/NMA/PCNA Guideline for the Prevention, Detection, Evaluation, and Management of High Blood Pressure in Adults. A report of the American College of Cardiology/American Heart Association Task Force on Clinical Practice Guidelines. *J. Am. Soc. Hypertens.* 12:238. 10.1016/j.jash.2018.01.004 29396104

[B32] Brookmeyer R. Gray S. Kawas C. (1998). Projections of Alzheimer’s disease in the United States and the public health impact of delaying disease onset. *Am. J. Public Health* 88 1337–1342. 10.2105/ajph.88.9.1337 9736873 PMC1509089

[B33] Broshek D. K. Kaushik T. Freeman J. R. Erlanger D. Webbe F. Barth J. T. (2005). Sex differences in outcome following sports-related concussion. *J. Neurosurg.* 102 856–863. 10.3171/jns.2005.102.5.0856 15926710

[B34] Brotfain E. Gruenbaum S. E. Boyko M. Kutz R. Zlotnik A. Klein M. (2016). Neuroprotection by estrogen and progesterone in traumatic brain injury and spinal cord injury. *Curr. Neuropharmacol.* 14 641–653. 10.2174/1570159x14666160309123554 26955967 PMC4981744

[B35] Brureau A. Zussy C. Delair B. Ogier C. Ixart G. Maurice T. (2013). Deregulation of hypothalamic-pituitary-adrenal axis functions in an Alzheimer’s disease rat model. *Neurobiol. Aging* 34 1426–1439. 10.1016/j.neurobiolaging.2012.11.015 23273603

[B36] Bryzgalova G. Gao H. Ahrén B. Zierath J. Galuska D. Steiler T. (2006). Evidence that oestrogen receptor-α plays an important role in the regulation of glucose homeostasis in mice: insulin sensitivity in the liver. *Diabetologia* 49 588–597. 10.1007/s00125-005-0105-3 16463047

[B37] Buckley R. F. Mormino E. C. Amariglio R. E. Properzi M. J. Rabin J. S. Lim Y. Y. (2018). Sex, amyloid, and APOE ε4 and risk of cognitive decline in preclinical Alzheimer’s disease: findings from three well-characterized cohorts. *Alzheimers Dement.* 14 1193–1203. 10.1016/j.jalz.2018.04.010 29803541 PMC6131023

[B38] Caracciolo B. Palmer K. Monastero R. Winblad B. Bäckman L. Fratiglioni L. (2008). Occurrence of cognitive impairment and dementia in the community: a 9-year-long prospective study. *Neurology* 70(19 Pt 2), 1778–1785. 10.1212/01.wnl.0000288180.21984.cb 18184916

[B39] Carter C. L. Resnick E. M. Mallampalli M. Kalbarczyk A. (2012). Sex and gender differences in Alzheimer’s disease: recommendations for future research. *J. Womens Health* 21 1018–1023. 10.1089/jwh.2012.3789 22917473

[B40] Casanova M. You L. Gaido K. W. Archibeque-Engle S. Janszen D. B. Heck H. D. A. (1999). Developmental effects of dietary phytoestrogens in Sprague-Dawley rats and interactions of genistein and daidzein with rat estrogen receptors alpha and beta in vitro. *Toxicol. Sci.* 51 236–244. 10.1093/toxsci/51.2.236 10543025

[B41] Cecconi S. Rucci N. Scaldaferri M. Masciulli M. Rossi G. Moretti C. (1999). Thyroid hormone effects on mouse oocyte maturation and granulosa cell aromatase activity. *Endocrinology* 140 1783–1788. 10.1210/en.140.4.1783 10098516

[B42] Chêne G. Beiser A. Au R. Preis S. R. Wolf P. A. Dufouil C. (2015). Gender and incidence of dementia in the Framingham Heart Study from mid-adult life. *Alzheimers Dement.* 11 310–320. 10.1016/j.jalz.2013.10.005 24418058 PMC4092061

[B43] Claxton A. Baker L. D. Wilkinson C. W. Trittschuh E. H. Chapman D. Watson G. (2013). Sex and ApoE genotype differences in treatment response to two doses of intranasal insulin in adults with mild cognitive impairment or Alzheimer’s disease. *J. Alzheimers Dis.* 35 789–797. 10.3233/jad-122308 23507773 PMC4144993

[B44] Cohen A. Pieper C. F. Brown A. J. Bastian L. A. (2006). Number of children and risk of metabolic syndrome in women. *J. Womens Health* 15 763–773. 10.1089/jwh.2006.15.763 16910908

[B45] Colcombe S. Kramer A. F. (2003). Fitness effects on the cognitive function of older adults: a meta-analytic study. *Psychol. Sci.* 14 125–130. 10.1111/1467-9280.t01-1-01430 12661673

[B46] Colcombe S. J. Erickson K. I. Scalf P. E. Kim J. S. Prakash R. McAuley E. (2006). Aerobic exercise training increases brain volume in aging humans. *J. Gerontol. A Biol. Sci. Med. Sci.* 61 1166–1170. 10.1093/gerona/61.11.1166 17167157

[B47] Colcombe S. J. Kramer A. F. Erickson K. I. Scalf P. McAuley E. Cohen N. J. (2004). Cardiovascular fitness, cortical plasticity, and aging. *Proc. Natl. Acad. Sci. U.S.A.* 101 3316–3321. 10.1073/pnas.0400266101 14978288 PMC373255

[B48] Colucci M. Cammarata S. Assini A. Croce R. Clerici F. Novello C. (2006). The number of pregnancies is a risk factor for Alzheimer’s disease. *Eur. J. Neurol.* 13 1374–1377. 17116223 10.1111/j.1468-1331.2006.01520.x

[B49] Convit A. (2005). Links between cognitive impairment in insulin resistance: an explanatory model.Neurobiol. *Aging* 26 31–35. 10.1016/j.neurobiolaging.2005.09.018 16246463

[B50] Corder E. H. Saunders A. M. Strittmatter W. J. Schmechel D. E. Gaskell P. C. Small G. (1993). Gene dose of apolipoprotein E type 4 allele and the risk of Alzheimer’s disease in late onset families. *Science* 261 921–923. 10.1126/science.8346443 8346443

[B51] Cui J. Shen Y. Li R. (2013). Estrogen synthesis and signaling pathways during aging: from periphery to brain. *Trends Mol. Med.* 19 197–209. 10.1016/j.molmed.2012.12.007 23348042 PMC3595330

[B52] Damoiseaux J. S. Seeley W. W. Zhou J. Shirer W. R. Coppola G. Karydas A. (2012). Gender modulates the APOE ε4 effect in healthy older adults: convergent evidence from functional brain connectivity and spinal fluid tau levels. *J. Neurosci.* 32 8254–8262. 10.1523/jneurosci.0305-12.2012 22699906 PMC3394933

[B53] Dassel K. B. Carr D. C. Vitaliano P. (2017). Does caring for a spouse with dementia accelerate cognitive decline? Findings from the health and retirement study. *Gerontologist* 57 319–328. 10.1093/geront/gnv148 26582383

[B54] Davis D. P. Douglas D. J. Smith W. Sise M. J. Vilke G. M. Holbrook T. L. (2006). Traumatic brain injury outcomes in pre-and post-menopausal females versus age-matched males. *J. Neurotrauma* 23 140–148. 10.1089/neu.2006.23.140 16503798

[B55] Day N. L. Floyd C. L. D’Alessandro T. L. Hubbard W. J. Chaudry I. H. (2013). 17 β-estradiol confers protection after traumatic brain injury in the rat and involves activation of g protein-coupled estrogen receptor 1. *J. Neurotrauma* 30 1531–1541. 10.1089/neu.2013.2854 23659385 PMC3751264

[B56] de Bruijn R. Ikram F. M. A. (2014). Cardiovascular risk factors and future risk of Alzheimer’s disease. *BMC Med.* 12:130. 10.1186/s12916-014-0130-5 25385322 PMC4226863

[B57] de la Rubia Ortí J. E. García-Pardo M. P. Drehmer E. Cantus D. S. Julián Rochina M. Aguilar M. (2018). Improvement of main cognitive functions in patients with alzheimer’s disease after treatment with coconut oil enriched mediterranean diet: a pilot study. *J. Alzheimers Dis.* 65 577–587. 10.3233/jad-180184 30056419

[B58] De Marchis G. M. Schaad C. Fung C. Beck J. Gralla J. Takala J. (2017). Gender-related differences in aneurysmal subarachnoid hemorrhage: a hospital based study. *Clin. Neurol. Neurosurg.* 157 82–87. 10.1016/j.clineuro.2017.04.009 28456071

[B59] del Ghianda S. Tonacchera M. Vitti P. (2014). Thyroid and menopause. *Climacteric* 17 225–234. 23998691 10.3109/13697137.2013.838554

[B60] den Heijer T. Vermeer S. Van Dijk E. Prins N. Koudstaal P. J. Hofman A. (2003). Type 2 diabetes and atrophy of medial temporal lobe structures on brain MRI. *Diabetologia* 46 1604–1610. 10.1007/s00125-003-1235-0 14595538

[B61] Den Ruijter H. M. Haitjema S. Asselbergs F. W. Pasterkamp G. (2015). Sex matters to the heart: a special issue dedicated to the impact of sex related differences of cardiovascular diseases. *Atherosclerosis* 241 205–207. 10.1016/j.atherosclerosis.2015.05.003 26003338

[B62] DeSantis S. M. Baker N. L. Back S. E. Spratt E. Ciolino J. D. Moran-Santa Maria M. (2011). Gender differences in the effect of early life trauma on hypothalamic–pituitary–adrenal axis functioning. *Depress. Anxiety* 28 383–392. 10.1002/da.20795 21328636 PMC3243643

[B63] Di Carlo A. Lamassa M. Baldereschi M. Inzitari M. Scafato E. Farchi G. (2007). CIND and MCI in the Italian elderly: frequency, vascular risk factors, progression to dementia. *Neurology* 68 1909–1916. 10.1212/01.wnl.0000263132.99055.0d 17536047

[B64] Ding F. Yao J. Rettberg J. R. Chen S. Brinton R. D. (2013). Early decline in glucose transport and metabolism precedes shift to ketogenic system in female aging and Alzheimer’s mouse brain: implication for bioenergetic intervention. *PLoS One* 8:e79977. 10.1371/journal.pone.0079977 24244584 PMC3823655

[B65] Dumitriu D. Rapp P. R. McEwen B. S. Morrison J. H. (2010). Estrogen and the aging brain: an elixir for the weary cortical network. *Ann. N. Y. Acad. Sci.* 1204 104–112. 10.1111/j.1749-6632.2010.05529.x 20738280 PMC2951002

[B66] Dunn N. Mullee M. Perry V. H. Holmes C. (2005). Association between dementia and infectious disease: evidence from a case-control study. *Alzheimer Dis. Assoc. Disord.* 19 91–94. 10.1097/01.wad.0000165511.52746.1f 15942327

[B67] Dykstra P. A. de Jong Gierveld J. (2004). Gender and marital-history differences in emotional and social loneliness among Dutch older adults. *Can. J. Aging* 23 141–155. 10.1353/cja.2004.0018 15334814

[B68] Echouffo-Tcheugui J. B. Conner S. C. Himali J. J. Maillard P. DeCarli C. S. Beiser A. S. (2018). Circulating cortisol and cognitive and structural brain measures: the framingham heart study. *Neurology* 91 e1961–e1970. 10.1212/wnl.0000000000006549 30355700 PMC6260201

[B69] Edland S. D. Rocca W. A. Petersen R. C. Cha R. H. Kokmen E. (2002). Dementia and Alzheimer disease incidence rates do not vary by sex in Rochester. Minn. *Arch. Neurol.* 59 1589–1593. 12374497 10.1001/archneur.59.10.1589

[B70] Edwards E. S. Sackett S. C. (2016). Psychosocial variables related to why women are less active than men and related health implications: supplementary issue: health disparities in women. *Clin Med. Insights Womens Health* 9(Suppl. 1), 47–56. 27398045 10.4137/CMWH.S34668 PMC4933535

[B71] Eggermont L. Swaab D. Luiten P. Scherder E. (2006). Exercise, cognition and Alzheimer’s disease: more is not necessarily better. *Neurosci. Biobehav. Rev.* 30 562–575. 10.1016/j.neubiorev.2005.10.004 16359729

[B72] Fallahpour M. Borell L. Luborsky M. Nygård L. (2016). Leisure-activity participation to prevent later-life cognitive decline: a systematic review. *Scand. J. Occup. Ther.* 23 162–197. 10.3109/11038128.2015.1102320 26586025

[B73] Farlow M. R. Lahiri D. Poirier J. Davignon J. Schneider L. Hui S. (1998). Treatment outcome of tacrine therapy depends on apolipoprotein genotype and gender of the subjects with Alzheimer’s disease. *Neurology* 50 669–677. 10.1212/wnl.50.3.669 9521254

[B74] Farmer J. Zhao X. Van Praag H. Wodtke K. Gage F. Christie B. (2004). Effects of voluntary exercise on synaptic plasticity and gene expression in the dentate gyrus of adult male Sprague–Dawley rats in vivo. *Neuroscience* 124 71–79. 10.1016/j.neuroscience.2003.09.029 14960340

[B75] Farrer L. A. Cupples L. A. Haines J. L. Hyman B. Kukull W. A. Mayeux R. (1997). Effects of age, sex, and ethnicity on the association between apolipoprotein E genotype and Alzheimer disease: a meta-analysis. *JAMA* 278 1349–1356. 10.1001/jama.278.16.1349 9343467

[B76] Feigin V. L. Rinkel G. J. Lawes C. M. Algra A. Bennett D. A. van Gijn J. (2005). Risk factors for subarachnoid hemorrhage: an updated systematic review of epidemiological studies. *Stroke* 36 2773–2780. 10.1161/01.str.0000190838.02954.e8 16282541

[B77] Ferretti M. T. Iulita M. F. Cavedo E. Chiesa P. A. Dimech A. S. Chadha A. S. (2018). Sex differences in Alzheimer disease—the gateway to precision medicine. *Nat. Rev. Neurol.* 14 457–469. 10.1038/s41582-018-0032-9 29985474

[B78] Fink G. Sumner B. E. Rosie R. Grace O. Quinn J. P. (1996). Estrogen control of central neurotransmission: effect on mood, mental state, and memory. *Cell Mol. Neurobiol.* 16 325–344. 10.1007/bf02088099 8818400 PMC11563142

[B79] Fisher D. W. Bennett D. A. Dong H. (2018). Sexual dimorphism in predisposition to Alzheimer’s disease. *Neurobiol. Aging.* 70 308–324. 10.1016/j.neurobiolaging.2018.04.004 29754747 PMC6368179

[B80] Fleisher A. Grundman M. Jack C. R. Petersen R. C. Taylor C. Kim H. T. (2005). Sex, apolipoprotein E ε4 status, and hippocampal volume in mild cognitive impairment. *Arch. Neurol.* 62 953–957. 15956166 10.1001/archneur.62.6.953

[B81] Fleminger S. Oliver D. Lovestone S. Rabe-Hesketh S. Giora A. (2003). Head injury as a risk factor for Alzheimer’s disease: the evidence 10 years on; a partial replication. *J. Neurol. Neurosurg. Psychiatry* 74 857–862. 10.1136/jnnp.74.7.857 12810767 PMC1738550

[B82] Fletcher A. Beevers D. Bulpitt C. Butler A. Coles E. Hunt D. (1988). Beta adrenoceptor blockade is associated with increased survival in male but not female hypertensive patients: a report from the DHSS hypertension care computing project (DHCCP). *J. Hum. Hypertens.* 2 219–227. 2907053

[B83] Foster T. C. (2012). Role of estrogen receptor alpha and beta expression and signaling on cognitive function during aging. *Hippocampus* 22 656–669. 10.1002/hipo.20935 21538657 PMC3704216

[B84] Fox M. Berzuini C. Knapp L. A. Glynn L. M. (2018). Women’s pregnancy life history and alzheimer’s risk: can immunoregulation explain the link? *Am. J. Alzheimers Dis. Other Demen.* 33 516–526. 10.1177/1533317518786447 30060670 PMC6442681

[B85] Fratiglioni L. Viitanen M. von Strauss E. Tontodonati V. Herlitz A. Winblad B. (1997). Very old women at highest risk of dementia and Alzheimer’s disease: incidence data from the Kungsholmen Project. Stockholm. *Neurology* 48 132–138. 10.1212/wnl.48.1.132 9008508

[B86] Ganguli M. Dodge H. H. Shen C. DeKosky S. T. (2004). Mild cognitive impairment, amnestic type: an epidemiologic study. *Neurology* 63 115–121. 10.1212/01.wnl.0000132523.27540.81 15249620

[B87] Garovic V. D. Bailey K. R. Boerwinkle E. Hunt S. C. Weder A. B. Curb D. (2010). Hypertension in pregnancy as a risk factor for cardiovascular disease later in life. *J. Hypertens.* 28 826–833. 10.1097/hjh.0b013e328335c29a 20087214 PMC2980863

[B88] Giridharan V. V. Thandavarayan R. A. Sato S. Ko K. M. Konishi T. (2011). Prevention of scopolamine-induced memory deficits by schisandrin B, an antioxidant lignan from Schisandra chinensis in mice. *Free Radic. Res.* 45 950–958. 10.3109/10715762.2011.571682 21615274

[B89] Giubilei F. Patacchioli F. Antonini G. Monti M. S. Tisei P. Bastianello S. (2001). Altered circadian cortisol secretion in Alzheimer’s disease: clinical and neuroradiological aspects. *J. Neurosci. Res.* 66 262–265. 10.1002/jnr.1219 11592122

[B90] Gleason C. E. Dowling N. M. Wharton W. Manson J. E. Miller V. M. Atwood C. S. (2015). Effects of hormone therapy on cognition and mood in recently postmenopausal women: findings from the randomized, controlled KEEPS–cognitive and affective study. *PLoS Med.* 12:e1001833. 10.1371/journal.pmed.1001833 26035291 PMC4452757

[B91] Glymour M. M. Manly J. J. (2008). Lifecourse social conditions and racial and ethnic patterns of cognitive aging. *Neuropsychol. Rev.* 18 223–254. 10.1007/s11065-008-9064-z 18815889

[B92] Goldstein J. M. Jerram M. Abbs B. Whitfield-Gabrieli S. Makris N. (2010). Sex differences in stress response circuitry activation dependent on female hormonal cycle. *J. Neurosci.* 30 431–438. 10.1523/jneurosci.3021-09.2010 20071507 PMC2827936

[B93] Gordon B. A. Rykhlevskaia E. I. Brumback C. R. Lee Y. Elavsky S. Konopack J. F. (2008). Neuroanatomical correlates of aging, cardiopulmonary fitness level, and education. *Psychophysiology* 45 825–838. 18627534 10.1111/j.1469-8986.2008.00676.x PMC5287394

[B94] Gordon J. L. Girdler S. S. Meltzer-Brody S. E. Stika C. S. Thurston R. C. Clark C. T. (2015). Ovarian hormone fluctuation, neurosteroids, and HPA axis dysregulation in perimenopausal depression: a novel heuristic model. *Am. J. Psychiatry* 172 227–236. 10.1176/appi.ajp.2014.14070918 25585035 PMC4513660

[B95] Gould E. Woolley C. S. Frankfurt M. McEwen B. S. (1990). Gonadal steroids regulate dendritic spine density in hippocampal pyramidal cells in adulthood. *J. Neurosci.* 10 1286–1291. 10.1523/jneurosci.10-04-01286.1990 2329377 PMC6570209

[B96] Grohé C. Kahlert S. Löbbert K. Stimpel M. Karas R. H. Vetter H. (1997). Cardiac myocytes and fibroblasts contain functional estrogen receptors 1. *FEBS Lett.* 416 107–112. 10.1016/s0014-5793(97)01179-4 9369244

[B97] Groot C. Hooghiemstra A. Raijmakers P. Van Berckel B. Scheltens P. Scherder E. (2016). The effect of physical activity on cognitive function in patients with dementia: a meta-analysis of randomized control trials. *Ageing Res. Rev.* 25 13–23. 10.1016/j.arr.2015.11.005 26607411

[B98] Gu Y. Nieves J. W. Stern Y. Luchsinger J. A. Scarmeas N. (2010). Food combination and Alzheimer disease risk: a protective diet. *Arch. Neurol.* 67 699–706. 20385883 10.1001/archneurol.2010.84 PMC3029147

[B99] Gu Y. Scarmeas N. (2011). Dietary patterns in Alzheimer’s disease and cognitive aging. *Curr. Alzheimer Res.* 8 510–519. 10.2174/156720511796391836 21605048 PMC3283139

[B100] Hajjar I. Quach L. Yang F. Chaves P. H. Newman A. B. Mukamal K. (2011). Hypertension, white matter hyperintensities, and concurrent impairments in mobility, cognition, and mood: the cardiovascular health study. *Circulation* 123 858–865. 10.1161/circulationaha.110.978114 21321150 PMC3081662

[B101] Håkansson K. Rovio E. L. Helkala A.-R. Vilska B. Winblad H. Soininen A. (2009). Association between mid-life marital status and cognitive function in later life: population based cohort study. *BMJ* 339:b2462. 10.1136/bmj.b2462 19574312 PMC2714683

[B102] Hall J. R. Wiechmann A. R. Johnson L. A. Edwards M. Barber R. C. Winter A. S. (2013). Biomarkers of vascular risk, systemic inflammation, and microvascular pathology and neuropsychiatric symptoms in alzheimer’s disease. *J. Alzheimers Dis.* 35 363–371. 10.3233/jad-122359 23403534 PMC3631280

[B103] Hamdan A. Barnes J. Mitchell P. (2014). Subarachnoid hemorrhage and the female sex: analysis of risk factors, aneurysm characteristics, and outcomes. *J. Neurosurg.* 121 1367–1373. 25216063 10.3171/2014.7.JNS132318

[B104] Hanamsagar R. Bilbo S. D. (2016). Sex differences in neurodevelopmental and neurodegenerative disorders: focus on microglial function and neuroinflammation during development. *J. Steroid Biochem. Mol. Biol.* 160 127–133. 10.1016/j.jsbmb.2015.09.039 26435451 PMC4829467

[B105] Harold D. Abraham R. Hollingworth P. Sims R. Gerrish A. Hamshere M. L. (2009). Genome-wide association study identifies variants at CLU and PICALM associated with Alzheimer’s disease. *Nat. Genet.* 41 1088–1093. 19734902 10.1038/ng.440 PMC2845877

[B106] Hashioka S. Miklossy J. Schwab C. Klegeris A. McGeer P. L. (2008). Adhesion of exogenous human microglia and THP-1 cells to amyloid plaques of postmortem Alzheimer’s disease brain. *J. Alzheimers Dis.* 14 345–352. 10.3233/jad-2008-14309 18599961

[B107] Hassing L. B. (2017). Gender differences in the association between leisure activity in adulthood and cognitive function in old age: a prospective longitudinal population-based study. *J. Gerontol. B* 10.1093/geronb/gbx170 [Epub ahead of print]. PMC6909435 29304225

[B108] Hayes J. P. Logue M. W. Sadeh N. Spielberg J. M. Verfaellie M. Hayes S. M. (2017). Mild traumatic brain injury is associated with reduced cortical thickness in those at risk for Alzheimer’s disease. *Brain* 140 813–825. 28077398 10.1093/brain/aww344 PMC6075586

[B109] Hebert L. E. Scherr P. A. McCann J. J. Beckett L. A. Evans D. A. (2001). Is the risk of developing Alzheimer’s disease greater for women than for men? *Am. J. Epidemiol.* 153 132–136. 11159157 10.1093/aje/153.2.132

[B110] Helmer C. Damon D. Letenneur L. Fabrigoule C. Barberger-Gateau P. Lafont S. (1999). Marital status and risk of Alzheimer’s disease A French population-based cohort study. *Neurology* 53 1953–1953. 10.1212/wnl.53.9.1953 10599764

[B111] Henderson V. W. John J. A. S. Hodis H. N. McCleary C. A. Stanczyk F. Z. Shoupe D. (2016). Cognitive effects of estradiol after menopause: a randomized trial of the timing hypothesis. *Neurology* 87 699–708. 10.1212/wnl.0000000000002980 27421538 PMC4999165

[B112] Heneka M. T. Carson M. J. El Khoury J. Landreth G. E. Brosseron F. Feinstein D. L. (2015). Neuroinflammation in Alzheimer’s disease. *Lancet Neurol.* 14 388–405. 25792098 10.1016/S1474-4422(15)70016-5 PMC5909703

[B113] Hillman C. H. Erickson K. I. Kramer A. F. (2008). Be smart, exercise your heart: exercise effects on brain and cognition. *Nat. Rev. Neurosci.* 9 58–65. 10.1038/nrn2298 18094706

[B114] Hodis H. N. Mack W. J. Henderson V. W. Shoupe D. Budoff M. J. Hwang-Levine J. (2016). Vascular effects of early versus late postmenopausal treatment with estradiol. *New Engl. J. Med.* 374 1221–1231. 10.1056/nejmoa1505241 27028912 PMC4921205

[B115] Hogervorst E. Clifford A. Stock J. Xin X. Bandelow S. (2012). Exercise to prevent cognitive decline and Alzheimer’s disease: for whom, when, what, and (most importantly) how much. *J. Alzheimers Dis. Parkinsonism* 2:e117.

[B116] Hohman T. J. Dumitrescu L. Barnes L. L. Thambisetty M. Beecham G. Kunkle B. (2018). Sex-Specific Association of Apolipoprotein E With Cerebrospinal Fluid Levels of Tau. *JAMA Neurol.* 75 989–998. 29801024 10.1001/jamaneurol.2018.0821 PMC6142927

[B117] Honig L. S. Tang M.-X. Albert S. Costa R. Luchsinger J. Manly J. (2003). Stroke and the risk of Alzheimer disease. *Arch. Neurol.* 60 1707–1712. 14676044 10.1001/archneur.60.12.1707

[B118] Horsburgh K. Macrae I. M. Carswell H. (2002). Estrogen is neuroprotective via an apolipoprotein E—dependent mechanism in a mouse model of global ischemia. *J. Cereb. Blood Flow Metab.* 22 1189–1195. 10.1097/01.wcb.0000037991.07114.4e 12368657

[B119] Hu D. Cao Y. He R. Han N. Liu Z. Miao L. (2012). Schizandrin, an antioxidant lignan from Schisandra chinensis, ameliorates Aβ 1–42-induced memory impairment in mice. *Oxid. Med. Cell. Longev.* 2012:721721. 10.1155/2012/721721 PMC3399599 22829961

[B120] Hu F. B. Stampfer M. J. Manson J. E. Rimm E. Colditz G. A. Rosner B. A. (1997). Dietary fat intake and the risk of coronary heart disease in women. *New Engl. J. Med.* 337 1491–1499. 9366580 10.1056/NEJM199711203372102

[B121] Huang C.-W. Lui C.-C. Chang W.-N. Lu C.-H. Wang Y.-L. Chang C.-C. (2009). Elevated basal cortisol level predicts lower hippocampal volume and cognitive decline in Alzheimer’s disease. *J. Clin. Neurosci.* 16 1283–1286. 10.1016/j.jocn.2008.12.026 19570680

[B122] Huang T. Lin B. M. Redline S. Curhan G. C. Hu F. B. Tworoger S. S. (2018). Type of menopause, age at menopause, and risk of developing obstructive sleep apnea in postmenopausal women. *Am. J. Epidemiol.* 187 1370–1379. 10.1093/aje/kwy011 29365014 PMC6030851

[B123] Humphries K. Izadnegahdar M. Sedlak T. Saw J. Johnston N. Schenck-Gustafsson K. (2017). Sex differences in cardiovascular disease-Impact on care and outcomes. *Front. Neuroendocrinol.* 46 46–70. 10.1016/j.yfrne.2017.04.001 28428055 PMC5506856

[B124] Huxley R. R. Peters S. A. Mishra G. D. Woodward M. (2015). Risk of all-cause mortality and vascular events in women versus men with type 1 diabetes: a systematic review and meta-analysis. *Lancet Diabetes Endocrinol.* 3 198–206. 10.1016/s2213-8587(14)70248-7 25660575

[B125] Iorga A. Cunningham C. M. Moazeni S. Ruffenach G. Umar S. Eghbali M. (2017). The protective role of estrogen and estrogen receptors in cardiovascular disease and the controversial use of estrogen therapy. *Biol. Sex Differ.* 8:33. 10.1186/s13293-017-0152-8 PMC5655818 29065927

[B126] Isaacson R. S. Ganzer C. A. Hristov H. Hackett K. Caesar E. Cohen R. (2018). The clinical practice of risk reduction for Alzheimer’s disease: A precision medicine approach. *Alzheimers Dement.* 14 1663–1673. 30446421 10.1016/j.jalz.2018.08.004 PMC6373477

[B127] Ishunina T. A. Fischer D. F. Swaab D. F. (2007). Estrogen receptor α and its splice variants in the hippocampus in aging and Alzheimer’s disease. *Neurobiol. Aging* 28 1670–1681. 10.1016/j.neurobiolaging.2006.07.024 17010478

[B128] Jeong E. J. Lee H. K. Lee K. Y. Jeon B. J. Kim D. H. Park J.-H. (2013). The effects of lignan-riched extract of Shisandra chinensis on amyloid-β-induced cognitive impairment and neurotoxicity in the cortex and hippocampus of mouse. *J. Ethnopharmacol.* 146 347–354. 10.1016/j.jep.2013.01.003 23333311

[B129] Johnson C. L. Rifkind B. M. Sempos C. T. Carroll M. D. Bachorik P. S. Briefel R. R. (1993). Declining serum total cholesterol levels among US adults: the national health and nutrition examination surveys. *JAMA* 269 3002–3008. 10.1001/jama.269.23.3002 8501842

[B130] Jones E. Eteiba W. Merz N. B. (2012). Cardiac syndrome X and microvascular coronary dysfunction. *Trends Cardiovasc. Med.* 22 161–168. 10.1016/j.tcm.2012.07.014 23026403 PMC3490207

[B131] Ju Y.-E. S. Lucey B. P. Holtzman D. M. (2014). Sleep and Alzheimer disease pathology—a bidirectional relationship. *Nat. Rev. Neurol.* 10 115–119. 10.1038/nrneurol.2013.269 24366271 PMC3979317

[B132] Ju Y.-E. S. McLeland J. S. Toedebusch C. D. Xiong C. Fagan A. M. Duntley S. P. (2013). Sleep quality and preclinical Alzheimer disease. *JAMA Neurol.* 70 587–593. 23479184 10.1001/jamaneurol.2013.2334 PMC3676720

[B133] Jungbauer A. Medjakovic S. (2014). Phytoestrogens and the metabolic syndrome. *J. Steroid Biochem. Mol. Biol.* 139 277–289. 10.1016/j.jsbmb.2012.12.009 23318879

[B134] Juutilainen A. Kortelainen S. Lehto S. Rönnemaa T. Pyörälä K. Laakso M. (2004). Gender difference in the impact of type 2 diabetes on coronary heart disease risk. *Diabetes Care* 27 2898–2904. 10.2337/diacare.27.12.2898 15562204

[B135] Kaczmarczyk M. M. Miller M. J. Freund G. G. (2012). The health benefits of dietary fiber: beyond the usual suspects of type 2 diabetes mellitus, cardiovascular disease and colon cancer. *Metabolism* 61 1058–1066. 10.1016/j.metabol.2012.01.017 22401879 PMC3399949

[B136] Kang J. H. Grodstein F. (2012). Postmenopausal hormone therapy, timing of initiation, APOE and cognitive decline. *Neurobiol. Aging* 33 1129–1137. 10.1016/j.neurobiolaging.2010.10.007 21122949 PMC3483632

[B137] Kannel W. B. Hjortland M. C. McNAMARA P. M. Gordon T. (1976). Menopause and risk of cardiovascular disease: the Framingham study. *Ann. Intern. Med.* 85 447–452. 970770 10.7326/0003-4819-85-4-447

[B138] Kantarci K. Lowe V. J. Lesnick T. G. Tosakulwong N. Bailey K. R. Fields J. A. (2016). Early postmenopausal transdermal 17β-estradiol therapy and amyloid-β deposition. *J. Alzheimers Dis.* 53 547–556. 10.3233/jad-160258 27163830 PMC4955514

[B139] Karp A. Kåreholt I. Qiu C. Bellander T. Winblad B. Fratiglioni L. (2004). Relation of education and occupation-based socioeconomic status to incident Alzheimer’s disease. *Am. J. Epidemiol.* 159 175–183. 10.1093/aje/kwh018 14718220

[B140] Katzman R. (1993). Education and the prevalence of dementia and Alzheimer’s disease. *Neurology* 43 13–20. 8423876 10.1212/wnl.43.1_part_1.13

[B141] Kehoe P. G. (2018). The coming of age of the angiotensin hypothesis in Alzheimer’s disease: progress toward disease prevention and treatment? *J. Alzheimers Dis.* 62 1443–1466. 10.3233/jad-171119 29562545 PMC5870007

[B142] Kelly D. F. McArthur D. L. Levin H. Swimmer S. Dusick J. R. Cohan P. (2006). Neurobehavioral and quality of life changes associated with growth hormone insufficiency after complicated mild, moderate, or severe traumatic brain injury. *J. Neurotrauma* 23 928–942. 10.1089/neu.2006.23.928 16774477

[B143] Kim J. Basak J. M. Holtzman D. M. (2009). The role of apolipoprotein E in Alzheimer’s disease. *Neuron* 63 287–303. 19679070 10.1016/j.neuron.2009.06.026 PMC3044446

[B144] Klein S. Pekosz A. Passaretti C. Anker M. Olukoya P. (2010). *Sex, Gender and Influenza* (Geneva: World Health Organization), 1–58.

[B145] Klose M. Juul A. Struck J. Morgenthaler N. Kosteljanetz M. Feldt-Rasmussen U. (2007). Acute and long-term pituitary insufficiency in traumatic brain injury: a prospective single-centre study. *Clin. Endocrinol.* 67 598–606. 10.1111/j.1365-2265.2007.02931.x 17880406

[B146] Koivisto K. Reinikainen K. Hanninen T. Vanhanen M. Helkala E. Mykkanen L. (1995). Prevalence of age-associated memory impairment in a randomly selected population from eastern Finland. *Neurology* 45 741–747. 10.1212/wnl.45.4.741 7723964

[B147] Kongable G. L. Lanzino G. Germanson T. P. Truskowski L. L. Alves W. M. Torner J. C. (1996). Gender-related differences in aneurysmal subarachnoid hemorrhage. *J. Neurosurg.* 84 43–48. 10.3171/jns.1996.84.1.0043 8613834

[B148] Kuiper G. G. Lemmen J. G. Carlsson B. Corton J. C. Safe S. H. Van P. (1998). Interaction of estrogenic chemicals and phytoestrogens with estrogen receptor β. *Endocrinology* 139 4252–4263. 10.1210/en.139.10.4252 9751507

[B149] Lagranha C. J. Silva T. L. A. Silva S. C. A. Braz G. R. F. da Silva A. I. Fernandes M. P. (2018). Protective effects of estrogen against cardiovascular disease mediated via oxidative stress in the brain. *Life Sci.* 192 190–198. 10.1016/j.lfs.2017.11.043 29191645

[B150] Larrieu S. Letenneur L. Orgogozo J. Fabrigoule C. Amieva H. Le Carret N. (2002). Incidence and outcome of mild cognitive impairment in a population-based prospective cohort. *Neurology* 59 1594–1599. 10.1212/01.wnl.0000034176.07159.f8 12451203

[B151] Laurin D. Verreault R. Lindsay J. MacPherson K. Rockwood K. (2001). Physical activity and risk of cognitive impairment and dementia in elderly persons. *Arch. Neurol.* 58 498–504. 11255456 10.1001/archneur.58.3.498

[B152] LeBlanc E. S. Janowsky J. Chan B. K. Nelson H. D. (2001). Hormone replacement therapy and cognition: systematic review and meta-analysis. *JAMA* 285 1489–1499. 11255426 10.1001/jama.285.11.1489

[B153] LeBlanc E. S. Kapphahn K. Hedlin H. Desai M. Parikh N. I. Liu S. (2017). Reproductive history and risk of type 2 diabetes mellitus in postmenopausal women: findings from the women’s health initiative. *Menopause* 24 64–72. 10.1097/gme.0000000000000714 27465714 PMC5477993

[B154] Leeners B. Geary N. Tobler P. N. Asarian L. (2017). Ovarian hormones and obesity. *Hum. Reprod. Update* 23 300–321. 10.1093/humupd/dmw045 28333235 PMC5850121

[B155] Li J.-Q. Tan L. Wang H.-F. Tan M.-S. Tan L. Xu W. (2016). Risk factors for predicting progression from mild cognitive impairment to Alzheimer’s disease: a systematic review and meta-analysis of cohort studies. *J. Neurol. Neurosurg. Psychiatry* 87 476–484. 10.1136/jnnp-2014-310095 26001840

[B156] Lines L. Sherif N. Wiener J. (2014). *Racial and Ethnic Disparities Among Individuals with Alzheimer’s Disease in the United States: A Literature Review.* Research Triangle Park, NC: RTI Press.

[B157] Liu C.-C. Kanekiyo T. Xu H. Bu G. (2013). Apolipoprotein E and Alzheimer disease: risk, mechanisms and therapy. *Nat. Rev. Neurol.* 9 106–118. 10.1038/nrneurol.2012.263 23296339 PMC3726719

[B158] Liu S. Willett W. C. Stampfer M. J. Hu F. B. Franz M. Sampson L. (2000). A prospective study of dietary glycemic load, carbohydrate intake, and risk of coronary heart disease in US women. *Am. J. Clin. Nutr.* 71 1455–1461. 10.1093/ajcn/71.6.1455 10837285

[B159] Livingston G. Sommerlad A. Orgeta V. Costafreda S. G. Huntley J. Ames D. (2017). Dementia prevention, intervention, and care. *Lancet* 390 2673–2734. 28735855 10.1016/S0140-6736(17)31363-6

[B160] Lovejoy J. Champagne C. De Jonge L. Xie H. Smith S. (2008). Increased visceral fat and decreased energy expenditure during the menopausal transition. *Int. J. Obes.* 32 949–958. 10.1038/ijo.2008.25 18332882 PMC2748330

[B161] Macgowan S. H. Wilcock G. K. Scott M. (1998). Effect of gender and apolipoprotein E genotype on response to anticholinesterase therapy in Alzheimer’s disease. *Int. J. Geriatr. Psychiatry* 13 625–630. 10.1002/(sici)1099-1166(199809)13:9<625::aid-gps835>3.0.co;2-2 9777427

[B162] Madrid-Valero J. J. Martínez-Selva J. M. Couto B. R. D. Sánchez-Romera J. F. Ordoñana J. R. (2017). Age and gender effects on the prevalence of poor sleep quality in the adult population. *Gac. Sanit.* 31 18–22. 10.1016/j.gaceta.2016.05.013 27474487

[B163] Maki P. M. (2013). The critical window hypothesis of hormone therapy and cognition: a scientific update on clinical studies. *Menopause* 20 695–709. 10.1097/GME.0b013e3182960cf8 23715379 PMC3780981

[B164] Mangold R. Sokoloff L. Conner E. Kleinerman J. Therman P.-O. G. Kety S. S. (1955). The effects of sleep and lack of sleep on the cerebral circulation and metabolism of normal young men. *J. Clin. Invest.* 34 1092–1100. 10.1172/jci103158 14392224 PMC438860

[B165] Manson J. E. Aragaki A. K. Rossouw J. E. Anderson G. L. Prentice R. L. LaCroix A. Z. (2017). Menopausal hormone therapy and long-term all-cause and cause-specific mortality: the Women’s Health Initiative randomized trials. *JAMA* 318 927–938. 28898378 10.1001/jama.2017.11217 PMC5728370

[B166] Marder K. Sano M. (2000). Estrogen to treat Alzheimer’s disease: too little, too late? So what’sa woman to do? *Neurology* 54 2035–2037. 10.1212/wnl.54.11.2035 10851358

[B167] Marks B. L. Madden D. J. Bucur B. Provenzale J. M. White L. E. Cabeza R. (2007). Role of aerobic fitness and aging on cerebral white matter integrity. *Ann. N. Y. Acad. Sci.* 1097 171–174. 10.1196/annals.1379.022 17413020

[B168] Mazure C. Jones M. D. P. (2015). Twenty years and still counting: including women as participants and studying sex and gender in biomedical research. *BMC Womens Health* 15:94. 10.1186/s12905-015-0251-9 26503700 PMC4624369

[B169] McEwen B. S. Akama K. T. Spencer-Segal J. L. Milner T. A. Waters E. M. (2012). Estrogen effects on the brain: actions beyond the hypothalamus via novel mechanisms. *Behav. Neurosci.* 126 4–16. 10.1037/a0026708 22289042 PMC3480182

[B170] McEwen B. S. Alves S. E. Bulloch K. Weiland N. G. (1997). Ovarian steroids and the brain: implications for cognition and aging. *Neurology* 48(5 Suppl. 7), 8S–15S. 10.1212/wnl.48.5_suppl_7.8s 9153161

[B171] Mendelsohn M. Karas R. H. (1999). The protective effects of estrogen on the cardiovascular system. *New Engl. J. Med.* 340 1801–1811. 10.1056/nejm199906103402306 10362825

[B172] Middleton L. E. Barnes D. E. Lui L. Y. Yaffe K. (2010). Physical activity over the life course and its association with cognitive performance and impairment in old age. *J. Am. Geriatr. Soc.* 58 1322–1326. 10.1111/j.1532-5415.2010.02903.x 20609030 PMC3662219

[B173] Miech R. Breitner J. Zandi P. Khachaturian A. Anthony J. Mayer L. (2002). Incidence of AD may decline in the early 90s for men, later for women: the cache county study. *Neurology* 58 209–218. 10.1212/wnl.58.2.209 11805246

[B174] Mielke M. M. Vemuri P. Rocca W. A. (2014). Clinical epidemiology of Alzheimer’s disease: assessing sex and gender differences. *Clin. Epidemiol.* 6 37–48. 24470773 10.2147/CLEP.S37929 PMC3891487

[B175] Miller V. M. Naftolin F. Asthana S. Black D. M. Brinton E. A. Budoff M. J. (2019). The kronos early estrogen prevention study (KEEPS): what have we learned? *Menopause* 10.1097/GME.0000000000001326 [Epub ahead of print]. 31453973 PMC6738629

[B176] Minett T. Classey J. Matthews F. E. Fahrenhold M. Taga M. Brayne C. (2016). Microglial immunophenotype in dementia with Alzheimer’s pathology. *J. Neuroinflamm.* 13:135. 10.1186/s12974-016-0601-z 27256292 PMC4890505

[B177] Möller-Leimkühler A. M. (2007). Gender differences in cardiovascular disease and comorbid depression. *Dialogues Clin. Neurosci.* 9 71–83. 17506227 10.31887/DCNS.2007.9.1/ammoeller PMC3181845

[B178] Mong J. A. Baker F. C. Mahoney M. M. Paul K. N. Schwartz M. D. Semba K. (2011). Sleep, rhythms, and the endocrine brain: influence of sex and gonadal hormones. *J. Neurosci.* 31 16107–16116. 10.1523/JNEUROSCI.4175-11.2011 22072663 PMC3249406

[B179] Monroe K. Murphy S. Kolonel L. Pike M. (2007). Prospective study of grapefruit intake and risk of breast cancer in postmenopausal women: the multiethnic cohort study. *Br. J. Cancer* 97 440. 10.1038/sj.bjc.6603880 17622247 PMC2360312

[B180] Morris M. Evans D. Bienias J. Tangney C. Wilson R. (2004). Dietary fat intake and 6-year cognitive change in an older biracial community population. *Neurology* 62 1573–1579. 10.1212/01.wnl.0000123250.82849.b6 15136684

[B181] Morris M. C. Tangney C. C. (2014). Dietary fat composition and dementia risk. *Neurobiol. Aging* 35 S59–S64. 10.1016/j.neurobiolaging.2014.03.038 24970568 PMC4107296

[B182] Morris M. C. Tangney C. C. Wang Y. Sacks F. M. Bennett D. A. Aggarwal N. T. (2015). MIND diet associated with reduced incidence of Alzheimer’s disease. *Alzheimers Dement.* 11 1007–1014. 10.1016/j.jalz.2014.11.009 25681666 PMC4532650

[B183] Mortimer J. Van Duijn C. Chandra V. Fratiglioni L. Graves A. Heyman A. (1991). Head trauma as a risk factor for Alzheimer’s disease: a collaborative re-analysis of case-control studies. *Int. J. Epidemiol.* 20(Suppl. 2), S28–S35. 1833351 10.1093/ije/20.supplement_2.s28

[B184] Mosconi L. Berti V. Guyara-Quinn C. McHugh P. Petrongolo G. Osorio R. S. (2017a). Perimenopause and emergence of an Alzheimer’s bioenergetic phenotype in brain and periphery. *PLoS One* 12:e0185926. 10.1371/journal.pone.0185926 29016679 PMC5634623

[B185] Mosconi L. Berti V. Quinn C. McHugh P. Petrongolo G. Varsavsky I. (2017b). Sex differences in Alzheimer risk brain imaging of endocrine vs chronologic aging. *Neurology* 89 1382–1390. 10.1212/WNL.0000000000004425 28855400 PMC5652968

[B186] Mosconi L. Murray J. Tsui W. Li Y. Davies M. Williams S. (2014). Mediterranean diet and magnetic resonance imaging-assessed brain atrophy in cognitively normal individuals at risk for Alzheimer’s disease. *J Prev. Alzheimers Dis.* 1 23–32. 25237654 PMC4165397

[B187] Mosconi L. Rahman A. Diaz I. Wu X. Scheyer O. Hristov H. (2018). Increased Alzheimer’s risk during the menopause transition: a 3-year longitudinal study. *PloS One* 13:e0207885. 10.1371/journal.pone.0207885 30540774 PMC6291073

[B188] Mulnard R. A. Cotman C. W. Kawas C. van Dyck C. H. Sano M. Doody R. (2000). Estrogen replacement therapy for treatment of mild to moderate Alzheimer disease: a randomized controlled trial. *JAMA* 283 1007–1015. 10697060 10.1001/jama.283.8.1007

[B189] Naderi V. Khaksari M. Abbasi R. Maghool F. (2015). Estrogen provides neuroprotection against brain edema and blood brain barrier disruption through both estrogen receptors α and β following traumatic brain injury. *Iran. J. Basic Med. Sci.* 18 138–144. 25810887 PMC4366724

[B190] Nagel G. Altenburg H. P. Nieters A. Boffetta P. Linseisen J. (2005). Reproductive and dietary determinants of the age at menopause in EPIC-Heidelberg. *Maturitas* 52 337–347. 10.1016/j.maturitas.2005.05.013 16011884

[B191] Napoles A. M. Chadiha L. Eversley R. Moreno-John G. (2010). Reviews: developing culturally sensitive dementia caregiver interventions: are we there yet? *Am. J. Alzheimers Dis. Other Demen.* 25 389–406. 10.1177/1533317510370957 20508244 PMC3581148

[B192] Nathan B. P. Barsukova A. G. Shen F. McAsey M. Struble R. G. (2004). Estrogen facilitates neurite extension via apolipoprotein E in cultured adult mouse cortical neurons. *Endocrinology* 145 3065–3073. 10.1210/en.2003-1707 15033916

[B193] Navaie-Waliser M. Feldman P. H. Gould D. A. Levine C. Kuerbis A. N. Donelan K. (2002). When the caregiver needs care: the plight of vulnerable caregivers. *Am. J. Public Health* 92 409–413. 10.2105/ajph.92.3.409 11867321 PMC1447090

[B194] Nebel R. A. Aggarwal N. T. Barnes L. L. Gallagher A. Goldstein J. M. Kantarci K. (2018). Understanding the impact of sex and gender in Alzheimer’s disease: a call to action. *Alzheimers Dement.* 14 1171–1183. 10.1016/j.jalz.2018.04.008 29907423 PMC6400070

[B195] Nemeroff C. B. (2007). Stress, menopause and vulnerability for psychiatric illness. *Exp. Rev. Neurother.* 7(Suppl.1), S11–S13. 10.1586/14737175.7.11s.S11 18039060

[B196] Neu S. C. Pa J. Kukull W. Beekly D. Kuzma A. Gangadharan P. (2017). Apolipoprotein E genotype and sex risk factors for Alzheimer disease: a meta-analysis. *JAMA Neurol.* 74 1178–1189. 10.1001/jamaneurol.2017.2188 28846757 PMC5759346

[B197] Nichols E. Szoeke C. E. Vollset S. E. Abbasi N. Abd-Allah F. Abdela J. (2019). Global, regional, and national burden of Alzheimer’s disease and other dementias, 1990–2016: a systematic analysis for the global burden of disease study 2016. *Lancet Neurol.* 18 88–106. 30497964 10.1016/S1474-4422(18)30403-4 PMC6291454

[B198] Nilsen J. Irwin R. W. Gallaher T. K. Brinton R. D. (2007). Estradiol in vivo regulation of brain mitochondrial proteome. *J. Neurosci.* 27 14069–14077. 10.1523/jneurosci.4391-07.2007 18094246 PMC6673510

[B199] Norton S. Matthews F. E. Barnes D. E. Yaffe K. Brayne C. (2014). Potential for primary prevention of Alzheimer’s disease: an analysis of population-based data. *Lancet Neurol.* 13 788–794. 10.1016/s1474-4422(14)70136-x 25030513

[B200] O’Hagan T. S. Wharton W. Kehoe P. G. (2012). Interactions between oestrogen and the renin angiotensin system-potential mechanisms for gender differences in Alzheimer’s disease. *Am. J. Neurodegener. Dis.* 1 266–279. 23383397 PMC3560469

[B201] Okereke O. I. Rosner B. A. Kim D. H. Kang J. H. Cook N. R. Manson J. E. (2012). Dietary fat types and 4-year cognitive change in community-dwelling older women. *Ann. Neurol.* 72 124–134. 10.1002/ana.23593 22605573 PMC3405188

[B202] Ott A. Breteler M. M. Harskamp F. V. Stijnen T. Hofman A. (1998). Incidence and risk of dementia: the Rotterdam Study. *Am. J. Epidemiol.* 147 574–580. 10.1093/oxfordjournals.aje.a009489 9521184

[B203] Paganini-Hill A. Henderson V. W. (1994). Estrogen deficiency and risk of Alzheimer’s disease in women. *Am. J. Epidemiol.* 140 256–261. 8030628 10.1093/oxfordjournals.aje.a117244

[B204] Park S. Harlow S. Zheng H. Karvonen-Gutierrez C. Thurston R. Ruppert K. (2017). Association between changes in oestradiol and follicle-stimulating hormone levels during the menopausal transition and risk of diabetes. *Diabet. Med.* 34 531–538. 10.1111/dme.13301 27973745 PMC5352524

[B205] Parker W. H. Jacoby V. Shoupe D. Rocca W. (2009). Effect of bilateral oophorectomy on women’s long-term health. *Womens Health* 5 565–576. 10.2217/whe.09.42 19702455

[B206] Pereira A. C. Huddleston D. E. Brickman A. M. Sosunov A. A. Hen R. McKhann G. M. (2007). An in vivo correlate of exercise-induced neurogenesis in the adult dentate gyrus. *Proc. Natl. Acad. Sci. U.S.A.* 104 5638–5643. 17374720 10.1073/pnas.0611721104 PMC1838482

[B207] Perry V. H. Cunningham C. Holmes C. (2007). Systemic infections and inflammation affect chronic neurodegeneration. *Nat. Rev. Immunol.* 7 161–167. 10.1038/nri2015 17220915

[B208] Peterson B. L. Won S. Geddes R. I. Sayeed I. Stein D. G. (2015). Sex-related differences in effects of progesterone following neonatal hypoxic brain injury. *Behav. Brain Res.* 286 152–165. 10.1016/j.bbr.2015.03.005 25746450

[B209] Phung T. K. T. Waltoft B. L. Laursen T. M. Settnes A. Kessing L. V. Mortensen P. B. (2010). Hysterectomy, oophorectomy and risk of dementia: a nationwide historical cohort study. *Dement. Geriatr. Cogn. Disord.* 30 43–50. 10.1159/000314681 20689282

[B210] Piccinelli M. Wilkinson G. (2000). Gender differences in depression: critical review. *Br. J. Psychiatry* 177 486–492. 10.1192/bjp.177.6.486 11102321

[B211] Pillar G. Malhotra A. Fogel R. Beauregard J. Schnall R. White D. P. (2000). Airway mechanics and ventilation in response to resistive loading during sleep: influence of gender. *Am. J. Respir. Crit. Care Med.* 162 1627–1632. 10.1164/ajrccm.162.5.2003131 11069787

[B212] Pilote L. Dasgupta K. Guru V. Humphries K. H. McGrath J. Norris C. (2007). A comprehensive view of sex-specific issues related to cardiovascular disease. *CMAJ* 176 S1–S44. 17353516 10.1503/cmaj.051455 PMC1817670

[B213] Pinquart M. Sörensen S. (2003). Differences between caregivers and noncaregivers in psychological health and physical health: a meta-analysis. *Psychol. Aging* 18 250–267. 10.1037/0882-7974.18.2.250 12825775

[B214] Podcasy J. L. Epperson C. N. (2016). Considering sex and gender in Alzheimer disease and other dementias. *Dialogues Clin. Neurosci.* 18 437–446. 28179815 10.31887/DCNS.2016.18.4/cepperson PMC5286729

[B215] Popovic R. M. White D. P. (1998). Upper airway muscle activity in normal women: influence of hormonal status. *J. Appl. Physiol.* 84 1055–1062. 10.1152/jappl.1998.84.3.1055 9480969

[B216] Prince M. Ali G.-C. Guerchet M. Prina A. M. Albanese E. Wu Y.-T. (2016). Recent global trends in the prevalence and incidence of dementia, and survival with dementia. *Alzheimers Res. Ther.* 8:23. 10.1186/s13195-016-0188-8 27473681 PMC4967299

[B217] Pucci G. Alcidi R. Tap L. Battista F. Mattace-Raso F. Schillaci G. (2017). Sex-and gender-related prevalence, cardiovascular risk and therapeutic approach in metabolic syndrome: a review of the literature. *Pharmacol. Res.* 120 34–42. 10.1016/j.phrs.2017.03.008 28300617

[B218] Rabi D. Khan N. Vallee M. Hladunewich M. Tobe S. Pilote L. (2008). Reporting on sex-based analysis in clinical trials of angiotensin-converting enzyme inhibitor and angiotensin receptor blocker efficacy. *Can. J. Cardiol.* 24 491–496. 10.1016/s0828-282x(08)70624-x 18548147 PMC2643194

[B219] Remely M. Lovrecic L. Garza A. Migliore L. Peterlin B. Milagro F. (2015). Therapeutic perspectives of epigenetically active nutrients. *Br. J. Pharmacol.* 172 2756–2768. 10.1111/bph.12854 25046997 PMC4439873

[B220] Rentz D. M. Weiss B. K. Jacobs E. G. Cherkerzian S. Klibanski A. Remington A. (2017). Sex differences in episodic memory in early midlife: impact of reproductive aging. *Menopause* 24 400–408. 10.1097/GME.0000000000000771 27824681 PMC5365356

[B221] Resnick S. M. Henderson V. W. (2002). Hormone therapy and risk of Alzheimer disease: a critical time. *JAMA* 288 2170–2172. 12413378 10.1001/jama.288.17.2170

[B222] Rettberg J. R. Yao J. Brinton R. D. (2014). Estrogen: a master regulator of bioenergetic systems in the brain and body. *Front. Neuroendocrinol.* 35 8–30. 10.1016/j.yfrne.2013.08.001 23994581 PMC4024050

[B223] Riant E. Waget A. Cogo H. Arnal J.-F. Burcelin R. Gourdy P. (2009). Estrogens protect against high-fat diet-induced insulin resistance and glucose intolerance in mice. *Endocrinology* 150 2109–2117. 10.1210/en.2008-0971 19164473

[B224] Riedel B. C. Thompson P. M. Brinton R. D. (2016). Age. APOE and sex: triad of risk of Alzheimer’s disease. *J. Steroid Biochem. Mol. Biol.* 160 134–147. 10.1016/j.jsbmb.2016.03.012 26969397 PMC4905558

[B225] Rietjens I. M. Louisse J. Beekmann K. (2017). The potential health effects of dietary phytoestrogens. *Br. J. Pharmacol.* 174 1263–1280. 10.1111/bph.13622 27723080 PMC5429336

[B226] Roberts R. Geda Y. Knopman D. Cha R. Pankratz V. Boeve B. (2012). The incidence of MCI differs by subtype and is higher in men: the mayo clinic study of aging. *Neurology* 78 342–351. 10.1212/WNL.0b013e3182452862 22282647 PMC3280046

[B227] Robinson D. P. Lorenzo M. E. Jian W. Klein S. L. (2011). Elevated 17β-estradiol protects females from influenza A virus pathogenesis by suppressing inflammatory responses. *PLoS Pathog.* 7:e1002149. 10.1371/journal.ppat.1002149 21829352 PMC3145801

[B228] Rocca W. Bower J. Maraganore D. Ahlskog J. Grossardt B. De Andrade M. (2007). Increased risk of cognitive impairment or dementia in women who underwent oophorectomy before menopause. *Neurology* 69 1074–1083. 10.1212/01.wnl.0000276984.19542.e6 17761551

[B229] Rocca W. A. Grossardt B. R. Shuster L. T. (2014). Oophorectomy, estrogen, and dementia: a 2014 update. *Mol. Cell. Endocrinol.* 389 7–12. 10.1016/j.mce.2014.01.020 24508665 PMC4040304

[B230] Rocca W. A. Grossardt B. R. Shuster L. T. Stewart E. A. (2012). Hysterectomy, oophorectomy, estrogen, and the risk of dementia. *Neurodegener. Dis.* 10 175–178. 10.1159/000334764 22269187 PMC3702015

[B231] Ropero A. B. Eghbali M. Minosyan T. Y. Tang G. Toro L. Stefani E. (2006). Heart estrogen receptor alpha: distinct membrane and nuclear distribution patterns and regulation by estrogen. *J. Mol. Cell Cardiol.* 41 496–510. 10.1016/j.yjmcc.2006.05.022 16876190

[B232] Rossouw J. E. Anderson G. L. Prentice R. L. LaCroix A. Z. Kooperberg C. Stefanick M. L. (2002). Risks and benefits of estrogen plus progestin in healthy postmenopausal women: principal results from the Women’s Health Initiative randomized controlled trial. *JAMA* 288 321–333. 10.1001/jama.288.3.321 12117397

[B233] Rothman S. M. Mattson M. P. (2010). Adverse stress, hippocampal networks, and Alzheimer’s disease. *Neuromolecular Med.* 12 56–70. 10.1007/s12017-009-8107-9 19943124 PMC2833224

[B234] Rubin T. G. Catenaccio E. Fleysher R. Hunter L. E. Lubin N. Stewart W. F. (2018). MRI-defined white matter microstructural alteration associated with soccer heading is more extensive in women than men. *Radiology* 289 478–486. 10.1148/radiol.2018180217 30063172 PMC6209057

[B235] Ruitenberg A. Ott A. van Swieten J. C. Hofman A. Breteler M. M. (2001). Incidence of dementia: does gender make a difference? *Neurobiol. Aging* 22 575–580. 10.1016/s0197-4580(01)00231-7 11445258

[B236] Ryan C. L. Siebens J. (2012). *Educational Attainment in the United States: 2009. Population Characteristics.* Current Population Reports. P20-566 Suitland, MD: US Census Bureau.

[B237] Ryan J. I Carrière, Carcaillon L. Dartigues J.-F. Auriacombe S. Rouaud O. (2014). Estrogen receptor polymorphisms and incident dementia: the prospective 3C study. *Alzheimers Dement.* 10 27–35. 10.1016/j.jalz.2012.12.008 23491264

[B238] Salloway S. Sperling R. Fox N. C. Blennow K. Klunk W. Raskind M. (2014). Two phase 3 trials of bapineuzumab in mild-to-moderate Alzheimer’s disease. *New Engl. J. Med.* 370 322–333. 10.1056/NEJMoa1304839 24450891 PMC4159618

[B239] Salpeter S. R. Cheng J. Thabane L. Buckley N. S. Salpeter E. E. (2009). Bayesian meta-analysis of hormone therapy and mortality in younger postmenopausal women. *Am. J. Med.* 122 1016.e11–1022.e11. 10.1016/j.amjmed.2009.05.021 19854329

[B240] Salpeter S. R. Walsh J. M. Greyber E. Ormiston T. M. Salpeter E. E. (2004). Mortality associated with hormone replacement therapy in younger and older women: a meta-analysis. *J. Gen. Intern. Med.* 19 791–804. 10.1111/j.1525-1497.2004.30281.x 15209595 PMC1492478

[B241] Samieri C. Grodstein F. Rosner B. A. Kang J. H. Cook N. R. Manson J. E. (2013). Mediterranean diet and cognitive function in older age: results from the women’s health study. *Epidemiology* 24 490–499. 10.1097/EDE.0b013e318294a065 23676264 PMC3674216

[B242] Sampedro F. Vilaplana E. De Leon M. J. Alcolea D. Pegueroles J. Montal V. (2015). APOE-by-sex interactions on brain structure and metabolism in healthy elderly controls. *Oncotarget* 6 26663–26674. 10.18632/oncotarget.5185 26397226 PMC4694943

[B243] Santos R. D. Waters D. D. Tarasenko L. Messig M. Jukema J. W. Ferrières J. (2009). Low-and high-density lipoprotein cholesterol goal attainment in dyslipidemic women: the lipid treatment assessment project (L-TAP) 2. *Am. Heart. J.* 158 860–866. 10.1016/j.ahj.2009.08.009 19853709

[B244] Scarmeas N. Anastasiou C. A. Yannakoulia M. (2018). Nutrition and prevention of cognitive impairment. *Lancet Neurol.* 17 1006–1015. 10.1016/s1474-4422(18)30338-7 30244829

[B245] Scarmeas N. Stern Y. Mayeux R. Luchsinger J. A. (2006). Mediterranean diet. Alzheimer disease, and vascular mediation. *Arch. Neurol.* 63 1709–1717. 17030648 10.1001/archneur.63.12.noc60109 PMC3024906

[B246] Scheyer O. Rahman A. Hristov H. Berkowitz C. Isaacson R. Brinton R. D. (2018). Female sex and Alzheimer’s risk: the menopause connection. *J. Prev. Alzheimers Dis.* 5 225–230. 10.14283/jpad.2018.34 30298180 PMC6198681

[B247] Schulz R. Beach S. R. (1999). Caregiving as a risk factor for mortality: the caregiver health effects study. *JAMA* 282 2215–2219. 10605972 10.1001/jama.282.23.2215

[B248] Schulze M. B. Liu S. Rimm E. B. Manson J. E. Willett W. C. Hu F. B. (2004). Glycemic index, glycemic load, and dietary fiber intake and incidence of type 2 diabetes in younger and middle-aged women. *Am. J. Clin. Nutr.* 80 348–356. 10.1093/ajcn/80.2.348 15277155

[B249] Seshadri S. Wolf P. Beiser A. Au R. McNulty K. White R. (1997). Lifetime risk of dementia and Alzheimer’s disease: the impact of mortality on risk estimates in the Framingham Study. *Neurology* 49 1498–1504. 10.1212/wnl.49.6.1498 9409336

[B250] Shen L. Melloni C. (2014). Representation of women in randomized clinical trials of cardiovascular disease prevention. *Curr. Cardiovasc. Risk Rep.* 8:390.

[B251] Shirato S. Swan B. A. (2010). Women and cardiovascular disease: an evidentiary review. *Medsurg. Nurs.* 19 282–286. 21189741

[B252] Shokri-Kojori E. Wang G.-J. Wiers C. E. Demiral S. B. Guo M. Kim S. W. (2018). β-Amyloid accumulation in the human brain after one night of sleep deprivation. *Proc. Natl. Acad. Sci. U.S.A.* 115 4483–4488. 10.1073/pnas.1721694115 29632177 PMC5924922

[B253] Shughrue P. J. Lane M. V. Merchenthaler I. (1997). Comparative distribution of estrogen receptor-α and-β mRNA in the rat central nervous system. *J. Comp. Neurol.* 388 507–525. 10.1002/(sici)1096-9861(19971201)388:4<507::aid-cne1>3.0.co;2-6 9388012

[B254] Shumaker S. A. Legault C. Rapp S. R. Thal L. Wallace R. B. Ockene J. K. (2003). Estrogen plus progestin and the incidence of dementia and mild cognitive impairment in postmenopausal women: the women’s health initiative memory study: a randomized controlled trial. *JAMA* 289 2651–2662. 12771112 10.1001/jama.289.20.2651

[B255] Slopien R. Wender-Ozegowska E. Rogowicz-Frontczak A. Meczekalski B. Zozulinska-Ziolkiewicz D. Jaremek J. D. (2018). Menopause and diabetes: EMAS clinical guide. *Maturitas* 117 6–10. 10.1016/j.maturitas.2018.08.009 30314563

[B256] Sobów T. M. Kloszewska I. (2003). Modulation of age at onset in late-onset sporadic Alzheimer’s disease by estrogen-related factors: the age of menopause and number of pregnancies. *Ger. J. Psychiatry* 6 49–55.

[B257] Soustiel J. F. Palzur E. Nevo O. Thaler I. Vlodavsky E. (2005). Neuroprotective anti-apoptosis effect of estrogens in traumatic brain injury. *J. Neurotrauma* 22 345–352. 10.1089/neu.2005.22.345 15785230

[B258] Sperling R. Mormino E. Johnson K. (2014). The evolution of preclinical Alzheimer’s disease: implications for prevention trials. *Neuron* 84 608–622. 10.1016/j.neuron.2014.10.038 25442939 PMC4285623

[B259] Spira A. P. Chen-Edinboro L. P. Wu M. N. Yaffe K. (2014). Impact of sleep on the risk of cognitive decline and dementia. *Curr. Opin. Psychiatry* 27 478–483. 10.1097/YCO.0000000000000106 25188896 PMC4323377

[B260] Sprecher K. E. Bendlin B. B. Racine A. M. Okonkwo O. C. Christian B. T. Koscik R. L. (2015). Amyloid burden is associated with self-reported sleep in nondemented late middle-aged adults. *Neurobiol. Aging* 36 2568–2576. 10.1016/j.neurobiolaging.2015.05.004 26059712 PMC4523445

[B261] Srivastava R. A. K. Krul E. S. Lin R. C. Schonfeld G. (1997). Regulation of lipoprotein metabolism by estrogen in inbred strains of mice occurs primarily by posttranscriptional mechanisms. *Mol. Cell. Biochem.* 173 161–168. 9278267 10.1023/a:1006896131186

[B262] Steiner M. Dunn E. Born L. (2003). Hormones and mood: from menarche to menopause and beyond. *J. Affect. Disord.* 74 67–83. 10.1016/s0165-0327(02)00432-9 12646300

[B263] Stephens M. A. C. Mahon P. B. McCaul M. E. Wand G. S. (2016). Hypothalamic–pituitary–adrenal axis response to acute psychosocial stress: effects of biological sex and circulating sex hormones. *Psychoneuroendocrinology* 66 47–55. 10.1016/j.psyneuen.2015.12.021 26773400 PMC4788592

[B264] Stern C. Munn Z. (2010). Cognitive leisure activities and their role in preventing dementia: a systematic review. *Int. J. Evid. Based Healthcare* 8 2–17. 10.1111/j.1744-1609.2010.00150.x 20923507

[B265] Stern Y. (2012). Cognitive reserve in ageing and Alzheimer’s disease. *Lancet Neurol.* 11 1006–1012. 10.1016/S1474-4422(12)70191-6 23079557 PMC3507991

[B266] Stern Y. Gurland B. Tatemichi T. K. Tang M. X. Wilder D. Mayeux R. (1994). Influence of education and occupation on the incidence of Alzheimer’s disease. *JAMA* 271 1004–1010. 10.1001/jama.271.13.1004 8139057

[B267] Straub R. H. Schradin C. (2016). Chronic inflammatory systemic diseases: an evolutionary trade-off between acutely beneficial but chronically harmful programs. *Evol. Med. Public Health* 2016 37–51. 10.1093/emph/eow001 26817483 PMC4753361

[B268] Struble R. Rosario E. Kircher M. Ludwig S. McAdamis P. Watabe K. (2003). Regionally specific modulation of brain apolipoprotein E in the mouse during the estrous cycle and by exogenous 17β estradiol. *Exp. Neurol.* 183 638–644. 10.1016/s0014-4886(03)00215-2 14552905

[B269] Sundermann E. E. Biegon A. Rubin L. H. Lipton R. B. Landau S. Maki P. M. (2017a). Does the female advantage in verbal memory contribute to underestimating Alzheimer’s disease pathology in women versus men? *J. Alzheimers Dis.* 56 947–957. 10.3233/jad-160716 28106548 PMC7644197

[B270] Sundermann E. E. Katz M. J. Lipton R. B. (2017b). Sex differences in the relationship between depressive symptoms and risk of amnestic mild cognitive impairment. *Am. J. Geriatr. Psychiatry* 25 13–22. 10.1016/j.jagp.2016.08.022 27986237 PMC5215465

[B271] Sundström A. Westerlund O. Mousavi-Nasab H. Adolfsson R. Nilsson L.-G. (2014). The relationship between marital and parental status and the risk of dementia. *Int. Psychogeriatr.* 26 749–757. 10.1017/S1041610213002652 24451183

[B272] Tan Z. S. Spartano N. L. Beiser A. S. DeCarli C. Auerbach S. H. Vasan R. S. (2016). Physical activity, brain volume, and dementia risk: the Framingham study. *J. Gerontol. A Biomed. Sci. Med. Sci.* 72 789–795. 10.1093/gerona/glw130 PMC6075525 27422439

[B273] Tan Z. S. Vasan R. S. (2009). Thyroid function and Alzheimer’s disease. *J. Alzheimers Dis.* 16 503–507. 19276542 10.3233/JAD-2009-0991

[B274] Thompson R. L. Lewis S. L. Murphy M. R. Hale J. M. Blackwell P. H. Acton G. J. (2004). Are there sex differences in emotional and biological responses in spousal caregivers of patients with Alzheimer’s disease? *Biol. Res. Nurs.* 5 319–330. 10.1177/1099800404263288 15068661

[B275] Troiano R. P. Berrigan D. Dodd K. W. Masse L. C. Tilert T. McDowell M. (2008). Physical activity in the United States measured by accelerometer. *Med. Sci. Sports Exerc.* 40 181–188. 18091006 10.1249/mss.0b013e31815a51b3

[B276] Um M. Y. Ahn J. Y. Kim S. Kim M. K. Ha T. Y. (2009). Sesaminol glucosides protect β-amyloid peptide-induced cognitive deficits in mice. *Biol. Pharm. Bull.* 32 1516–1520. 10.1248/bpb.32.1516 19721225

[B277] Ungar L. Altmann A. Greicius M. D. (2014). Apolipoprotein E, gender, and Alzheimer’s disease: an overlooked, but potent and promising interaction. *Brain Imaging Behav.* 8 262–273. 10.1007/s11682-013-9272-x 24293121 PMC4282773

[B278] Uryu K. Chen X.-H. Martinez D. Browne K. D. Johnson V. E. Graham D. I. (2007). Multiple proteins implicated in neurodegenerative diseases accumulate in axons after brain trauma in humans. *Exp. Neurol.* 208 185–192. 10.1016/j.expneurol.2007.06.018 17826768 PMC3979356

[B279] Van Praag H. (2008). Neurogenesis and exercise: past and future directions. *Neuromolecular Med.* 10 128–140. 10.1007/s12017-008-8028-z 18286389

[B280] Van Praag H. (2009). Exercise and the brain: something to chew on. *Trends Neurosci.* 32 283–290. 10.1016/j.tins.2008.12.007 19349082 PMC2680508

[B281] Van Praag H. Christie B. R. Sejnowski T. J. Gage F. H. (1999). Running enhances neurogenesis, learning, and long-term potentiation in mice. *Proc. Natl. Acad. Sci. U.S.A.* 96 13427–13431. 10.1073/pnas.96.23.13427 10557337 PMC23964

[B282] Van Steenoven I. Aarsland D. Weintraub D. Londos E. Blanc F. Van der Flier W. M. (2016). Cerebrospinal fluid Alzheimer’s disease biomarkers across the spectrum of Lewy body diseases: results from a large multicenter cohort. *J. Alzheimers Dis.* 54 287–295. 10.3233/jad-160322 27567832 PMC5535729

[B283] Vemuri P. Lesnick T. (in press). Effect of lifestyle activities on AD biomarkers and cognition. *Ann. Neurol.* 72 730–738. 23280791 10.1002/ana.23665 PMC3539211

[B284] Verdelho A. Madureira S. Moleiro C. Ferro J. M. O’Brien J. T. Poggesi A. (2013). Depressive symptoms predict cognitive decline and dementia in older people independently of cerebral white matter changes: the LADIS study. *J. Neurol. Neurosurg. Psychiatry* 84 1250–1254. 10.1136/jnnp-2012-304191 23715914

[B285] Verhoef M. Love E. J. Rose S. A. (1993). Women’s social roles and their exercise participation. *Women Health* 19 15–29. 10.1300/j013v19n04_02 1295266

[B286] Villegas-Llerena C. Phillips A. Garcia-Reitboeck P. Hardy J. Pocock J. M. (2016). Microglial genes regulating neuroinflammation in the progression of Alzheimer’s disease. *Curr. Opin. Neurobiol.* 36 74–81. 10.1016/j.conb.2015.10.004 26517285

[B287] Vina J. Lloret A. (2010). Why women have more Alzheimer’s disease than men: gender and mitochondrial toxicity of amyloid-β peptide. *J. Alzheimers Dis.* 20 S527–S533. 10.3233/JAD-2010-100501 20442496

[B288] Vina J. Sastre J. Pallardo F. Gambini J. Borras C. (2006). Role of mitochondrial oxidative stress to explain the different longevity between genders. Protective effect of estrogens. *Free Radic. Res.* 40 1359–1365. 10.1080/10715760600952851 17090425

[B289] Wang H.-X. Xu W. Pei J.-J. (2012). Leisure activities, cognition and dementia. *Biochim. Biophys. Acta Mol. Basis Dis.* 1822 482–491. 10.1016/j.bbadis.2011.09.002 21930203

[B290] Wang P. Liao S. Liu R. Liu C. Chao H. Lu S. (2000). Effects of estrogen on cognition, mood, and cerebral blood flow in AD A controlled study. *Neurology* 54 2061–2066. 10.1212/wnl.54.11.2061 10851363

[B291] Wei Y.-C. George N. I. Chang C.-W. Hicks K. A. (2017). Assessing sex differences in the risk of cardiovascular disease and mortality per increment in systolic blood pressure: a systematic review and meta-analysis of follow-up studies in the United States. *PLoS One* 12:e0170218. 10.1371/journal.pone.0170218 28122035 PMC5266379

[B292] Westphal S. A. (2008). Obesity, abdominal obesity, and insulin resistance. *Clin. Cornerstone* 9 23–31. 10.1016/s1098-3597(08)60025-3 19046737

[B293] Wild S. Roglic G. Green A. Sicree R. King H. (2004). Global prevalence of diabetes: estimates for the year 2000 and projections for 2030. *Diabetes Care* 27 1047–1053. 10.2337/diacare.27.5.1047 15111519

[B294] Willey J. Z. Gardener H. Caunca M. R. Moon Y. P. Dong C. Cheung Y. K. (2016). Leisure-time physical activity associates with cognitive decline: the northern manhattan study. *Neurology* 86 1897–1903. 10.1212/WNL.0000000000002582 27009261 PMC4873686

[B295] Wilson R. S. Barnes L. DeLeon C. M. Aggarwal N. Schneider J. Bach J. (2002). Depressive symptoms, cognitive decline, and risk of AD in older persons. *Neurology* 59 364–370. 10.1212/wnl.59.3.364 12177369

[B296] Woolley C. S. Gould E. Frankfurt M. McEwen B. S. (1990). Naturally occurring fluctuation in dendritic spine density on adult hippocampal pyramidal neurons. *J. Neurosci.* 10 4035–4039. 10.1523/jneurosci.10-12-04035.1990 2269895 PMC6570039

[B297] Woolley C. S. McEwen B. S. (1992). Estradiol mediates fluctuation in hippocampal synapse density during the estrous cycle in the adult rat. *J. Neurosci.* 12 2549–2554. 10.1523/jneurosci.12-07-02549.1992 1613547 PMC6575846

[B298] Wyss-Coray T. (2006). Inflammation in Alzheimer disease: driving force, bystander or beneficial response? *Nat. Med.* 12 1005–1015. 16960575 10.1038/nm1484

[B299] Wyss-Coray T. Mucke L. (2002). Inflammation in neurodegenerative disease—a double-edged sword. *Neuron* 35 419–432. 10.1016/s0896-6273(02)00794-8 12165466

[B300] Xie L. Kang H. Xu Q. Chen M. J. Liao Y. Thiyagarajan M. (2013). Sleep drives metabolite clearance from the adult brain. *Science* 342 373–377. 10.1126/science.1241224 24136970 PMC3880190

[B301] Yaffe K. Barnes D. Nevitt M. Lui L.-Y. Covinsky K. (2001). A prospective study of physical activity and cognitive decline in elderly women: women who walk. *Arch. Intern. Med.* 161 1703–1708. 11485502 10.1001/archinte.161.14.1703

[B302] Yaffe K. Blackwell T. Gore R. Sands L. Reus V. Browner W. S. (1999). Depressive symptoms and cognitive decline in nondemented elderly women: a prospective study. *Arch. Gen. Psychiatry* 56 425–430. 10232297 10.1001/archpsyc.56.5.425

[B303] Yaffe K. Haan M. Byers A. Tangen C. Kuller L. (2000). Estrogen use. APOE, and cognitive decline: evidence of gene–environment interaction. *Neurology* 54 1949–1954. 10.1212/wnl.54.10.1949 10822435

[B304] Yaffe K. Lindquist K. Sen S. Cauley J. Ferrell R. Penninx B. (2009). Estrogen receptor genotype and risk of cognitive impairment in elders: findings from the Health ABC study. *Neurobiol. Aging* 30 607–614. 10.1016/j.neurobiolaging.2007.08.003 17889406 PMC2826192

[B305] Yen S. (1986). *Reproductive Endocrinology.* Philadelphia: WB Saunders Co.

[B306] Zandi P. P. Carlson M. C. Plassman B. L. Welsh-Bohmer K. A. Mayer L. S. Steffens D. C. (2002). Hormone replacement therapy and incidence of Alzheimer disease in older women: the cache county study. *JAMA* 288 2123–2129. 12413371 10.1001/jama.288.17.2123

[B307] Zhao L. Mao Z. Woody S. K. Brinton R. D. (2016). Sex differences in metabolic aging of the brain: insights into female susceptibility to Alzheimer’s disease. *Neurobiol. Aging* 42 69–79. 10.1016/j.neurobiolaging.2016.02.011 27143423 PMC5644989

[B308] Zissimopoulos J. Crimmins E. Clair P. S. (2015). The value of delaying Alzheimer’s disease onset. *Forum Health Econ. Policy* 18 25–39. 10.1515/fhep-2014-0013 27134606 PMC4851168

[B309] Zissimopoulos J. M. Barthold D. Brinton R. D. Joyce G. (2017). Sex and race differences in the association between statin use and the incidence of Alzheimer disease. *JAMA Neurol.* 74 225–232. 10.1001/jamaneurol.2016.3783 27942728 PMC5646357

